# NR4A1 as a potential therapeutic target in colon adenocarcinoma: a computational analysis of immune infiltration and drug response

**DOI:** 10.3389/fgene.2023.1181320

**Published:** 2023-07-26

**Authors:** Mei Li, Zhongyi Zhang, Mingzhou Li, Zhe Chen, Weizhu Tang, Xiang Cheng

**Affiliations:** ^1^ Department of Oncology, The First Affiliated Hospital of the Hubei Three Gorges Polytechnic, Yiling Hospital of Yichang, Yichang, Hubei, China; ^2^ The Second Affiliated Hospital of Guilin Medical University, Guilin Medical University, Guilin, Guangxi, China; ^3^ Nanxishan Hospital of Guangxi Zhuang Autonomous Region, Guilin, Guangxi, China; ^4^ School of Information and Communication, Guilin University of Electronic Technology, Guilin, Guangxi, China; ^5^ Taikang Ningbo Hospital, Ningbo, China; ^6^ Union Hospital, Tongji Medical College, Huazhong University of Science and Technology, Wuhan, China

**Keywords:** colon adenocarcinoma, immune infiltration, single cell sequencing, predictive model, precision medicine

## Abstract

**Background:** Colon adenocarcinoma (COAD) is a common malignancy with high morbidity and mortality rates. The immune system plays a crucial role in CRC development and progression, making it a potential therapeutic target. In this study, we analyzed transcriptomic data from CRC patients to investigate immune infiltration and identify potential therapeutic targets.

**Method and results:** we used CIBERSORT to analyze the immune infiltration in COAD samples and found that the high infiltration of M2 macrophages and neutrophils was associated with poor prognosis. Next, we identified NR4A1 as a potential therapeutic target based on its protective effect in two predict models. Using cancer therapeutics response analysis, we found that high expression levels of NR4A1 were sensitive to OSI-930, a tyrosine kinase inhibitor with anti-tumor effects.

**Conclusion:** Our findings suggest that targeting NR4A1 with OSI-930 may be a promising therapeutic strategy for COAD patients with high levels of immune infiltration. However, further studies are needed to investigate the clinical efficacy of this approach.

## 1 Introduction

Colon adenocarcinoma (COAD) is a malignant tumor that arises from the glandular epithelium lining the colon, which is a part of the large intestine. It is the third most common cancer and the second leading cause of cancer-related deaths worldwide. The 5-year survival rate for colon adenocarcinoma patients remains relatively low, ranging from 90% for localized disease to 14% for distant metastases, despite treatment advancements such as surgery, chemotherapy, and radiation therapy. Early detection and treatment, particularly with surgery, are critical for improving survival rates (Galon et al., 2006; Siegel et al., 2020).

Research is ongoing to identify biomarkers and risk factors that may improve early detection and treatment options for colon adenocarcinoma patients, as well as to develop new targeted therapies and immunotherapies for advanced disease. Recent research has suggested that the immune system plays a crucial role in the development and progression of colon adenocarcinoma, and the infiltration of immune cells, such as dendritic cells, into the tumor microenvironment may influence patient outcomes (Galon et al., 2006; Fridman et al., 2012). Studies have shown that the density and function of immune cells in the tumor microenvironment can affect the efficacy of cancer treatments and predict patient outcomes (Pages et al., 2005; Galon et al., 2006; Fridman et al., 2012). Therefore, understanding the immune infiltration in the tumor microenvironment could provide insights into the development of effective cancer treatments for colon adenocarcinoma patients.

However, Studying the role of immune infiltration in colon adenocarcinoma can be challenging due to the complexity of the tumor microenvironment, which involves various interacting immune and stromal cells. While immune infiltrates may predict clinical outcomes in some studies, it remains unclear how this information can guide clinical decision-making, including treatment selection. Further research is necessary to translate immune infiltration into actionable clinical information.

Single-cell RNA sequencing (scRNA-seq) is a powerful tool to study the complexity of the tumor microenvironment in colon adenocarcinoma. scRNA-seq allows profiling of gene expression in individual cells providing new insights into the role of the immune system in tumor development and biomarkers for early detection and targeted therapies (Hanzelmann et al., 2013; Zhang et al., 2019; Korber et al., 2020).

The present study seeks to address the challenges by utilizing scRNA-seq in combination with bulk RNA sequencing data. Specifically, the integration of scRNA-seq data with bulk RNA sequencing data enables the identification of cell specific gene expression profiles, thereby providing a more comprehensive understanding of the heterogeneous tumor microenvironment. Additionally, this study aims to identify potential biomarkers for survival prediction and to develop new targeted therapies for COAD, with the ultimate goal of providing clinically relevant information that can inform treatment decisions.

## 2 Materials and methods

### 2.1 Data collection

Data for this study were obtained from the Gene Expression Omnibus (GEO) and The Cancer Genome Atlas (TCGA) databases. The GEO dataset (GSE166555) contains scRNA-seq data from colon adenocarcinoma tumor tissue samples and normal colon tissue samples. The TCGA dataset contains genomic and clinical data from colon adenocarcinoma patients.

Two GEO dataset (GSE39582 and GSE87211) contains genomic and clinical data from colon adenocarcinoma patients were obtained for external validation.

Protein staining results were obtained from human protein atlas.

### 2.2 Bulk RNA data processing

The gene length was obtained from the GFF3 file using the Bioconductor package “GenomicFeatures” in R (Zhu et al., 2010; Lawrence et al., 2013). The gene count was obtained from the raw RNA sequencing data using the featureCounts function in the Subread package. The counts were then normalized to transcripts per million (TPM) using the formula:

$$TPM=\left(gene\_count\;/\;gene\_length\right)\;*\;\left(1,000,000\;/\;total\_counts\right)$$

Bulk RNA data processing and analysis were performed using R software. The limma package was used for normalization and differential gene expression analysis (Ritchie et al., 2015). Fold change >2 or < −2 (|log2FC > 1|), adjusted *p*-value <0.05 were set.

These genes were subjected to pathway enrichment analysis using the Gene Ontology database (Mi et al., 2019) and the Kyoto Encyclopedia of Genes and Genomes (KEGG) database (Kanehisa and Goto, 2000). The significantly enriched pathways were identified based on a *p*-value threshold of 0.05.

### 2.3 Prognostic significance analysis of clinic features

We used the rms R package to integrate data on survival time, survival status, and five clinic features (Age, Gender, Stage, Relapse, Histological type). We used the Cox proportional hazards regression method to build a nomogram and evaluated the prognostic significance of these features in 427 samples.

### 2.4 Immune analysis of bulk RNA

CIBERSORT analysis was then performed using the CIBERSORT package in R (Hao et al., 2021). The LM22 gene signature matrix was used as a reference to identify the relative proportions of 22 different immune cell types in each sample (Newman et al., 2015). To ensure the reliability of the CIBERSORT results, only samples with a CIBERSORT *p*-value <0.05 were considered in the subsequent analysis. The resulting immune cell proportions were then used for downstream analysis, including survival analysis and correlation analysis with clinical variables.

### 2.5 ScRNA data processing

To analyze the single cell sequencing data of colon adenocarcinoma, we used the Seurat package in R (Stuart et al., 2019). Seurat is a widely used computational tool for single cell RNA sequencing analysis, which provides functions for quality control, data normalization, dimensionality reduction, clustering, marker gene finding, and visualization.

After obtaining the single cell RNA sequencing data, quality control was performed using the Seurat functions “CreateSeuratObject” and “FilterCells”. Cells with a low number of genes expressed (<200) and high percentage of mitochondrial genes (>5%) were removed. The remaining cells were then normalized and scaled using the “NormalizeData” and “ScaleData” functions, respectively.

Dimensionality reduction was performed using the “RunPCA”, RunTSNE”, and “RunUMAP” functions to reduce the high-dimensional data into a two-dimensional space for visualization. Clustering analysis was performed using the “FindClusters” function, and cell types were identified using the “FindAllMarkers” function to find the marker genes for each cluster.

Visualization of the clustering results was performed using the “DimPlot” and “FeaturePlot” functions, which generate scatter plots of the cells and expression levels of marker genes for each cluster, respectively.

To annotate the clusters obtained from Seurat analysis of single cell RNA sequencing data, we used the singleR package in R (Aran et al., 2019). singleR is a computational tool that provides a reference-based approach to annotate the cell types in scRNA-seq data.

### 2.6 Survival analysis of immune types

To analyze the effect of different immune cell proportions on the survival rate of colon adenocarcinoma patients, we used the Kaplan-Meier (KM) curve analysis. KM curve analysis is a statistical method used to estimate the probability of survival over time for different groups of patients based on their clinical or molecular characteristics.

We obtained the patient data from TCGA database and divided them into two groups based on each immune cell proportions: high proportion group and low proportion group. The cutoff value for defining high and low proportion was determined using the median proportion.

The KM curve analysis was performed using the “survival” package in R. The “Surv” function was used to create a survival object, which includes the survival time and censoring status for each patient. The “survfit” function was used to estimate the survival probability over time for each group, and the “logrank” test was used to compare the survival curves between the two groups.

To assess the significance of the difference in survival between the two groups, we calculated the hazard ratio (HR) and 95% confidence interval (CI) using the “coxph” function in the “survival” package.

Genes associated with the target immune cell type were obtained from scRNA-seq for further analysis.

### 2.7 Predict model based on neutrophils cells and macrophages M2

Genes with the annotation as neutrophils cells and macrophages M2, were abstracted from scRNA-seq clusters. To identify the genes that have a significant impact on survival in colon adenocarcinoma patients, we performed a univariate Cox regression analysis using the “survival” package in R (Simon et al., 2011). In this analysis, we tested the association between the expression levels of each gene and the survival time of patients.

To select the genes with the most significant impact on survival, we used a *p*-value cutoff of 0.05. Genes with a *p*-value lower than 0.05 were considered significant and selected for the least absolute shrinkage and selection operator (lasso) Cox regression.

Lasso Cox regression is a type of regularized regression that can handle high-dimensional data and select the most important predictors while shrinking the coefficients of less important predictors to zero.

The “glmnet” package in R was used to perform the lasso Cox regression analysis. The optimal value of the penalty parameter lambda was chosen using the 10-fold cross-validation method.

The performance of the predictive model was evaluated using the concordance index (C-index), which measures the accuracy of the model in predicting the survival outcome. The C-index ranges from 0.5 (random prediction) to 1 (perfect prediction).

### 2.8 Chemical compounds sensitivities

To analyze the correlation between gene expression and chemical compound sensitivities, we used the Cancer Therapeutics Response Portal (CTRP) v2 database (Rees et al., 2016). CTRPV2 is a web-based platform that integrates gene expression and drug sensitivity data from the GDSC database to identify potential biomarkers for drug sensitivity.

We accessed the CTRP v2 online tool (https://portals.broadinstitute.org/ctrp/), which provides a user-friendly interface for exploring and analyzing the CTRP v2 data. We input the TPM values of the genes of interest and the IC50 values of the chemical compounds of interest into CTRPV2 and obtained the correlation coefficient (r) for each gene-compound pair.

The tool provides a Z-score for each gene-compound pair, which represents the number of standard deviations from the mean expression level for that gene in response to that compound. We set a Z-score of 1.5 as the cutoff for statistically significant correlation between gene expression and chemical compound sensitivity.

## 3 Result

### 3.1 Data collection

In this study, a total of 428 COAD samples from the TCGA database were included. The clinicopathological characteristics of the patients are summarized in Table 1; Supplementary Figure S1. The patients had a median age of 68 years (range: 31–90 years), with a slight predominance of males (53.86%). The majority of patients had stage II (38.87%) or stage III (28.8%) disease at diagnosis. Patients with missing clinical information or survival status were excluded from the analysis. The exclusion criteria for TCGA data were as follows: 1) Patients with missing clinical information, 2) patients with a history of other malignancies, 3) patients with an unmentioned histological type and 4) patients who had a survival time of 0 days.

**TABLE 1:** The clinicopathological characteristics of the 427 enrolled patients.

Characteristics	Deceased (*N* = 336)	Living (*N* = 91)	Total (*N* = 427)
Age			
Mean ± SD	65.68 ± 12.64	68.67 ± 13.23	66.31 ± 12.81
Median [min-max]	67.00 [31.00,90.00]	71.00 [34.00,90.00]	68.00 [31.00,90.00]
Gender			
Female	158 (37.00%)	39 (9.13%)	197 (46.14%)
Male	178 (41.69%)	52 (12.18%)	230 (53.86%)
Stage			
Stage I	55 (12.88%)	17 (3.98%)	72 (16.86%)
Stage IA	1 (0.23%)	0 (0.0e + 0%)	1 (0.23%)
Stage II	24 (5.62%)	5 (1.17%)	29 (6.79%)
Stage IIA	98 (22.95%)	28 (6.56%)	126 (29.51%)
Stage IIB	7 (1.64%)	3 (0.70%)	10 (2.34%)
Stage IIC	1 (0.23%)	0 (0.0e+0%)	1 (0.23%)
Stage III	13 (3.04%)	4 (0.94%)	17 (3.98%)
Stage IIIA	5 (1.17%)	3 (0.70%)	8 (1.87%)
Stage IIIB	50 (11.71%)	8 (1.87%)	58 (13.58%)
Stage IIIC	30 (7.03%)	10 (2.34%)	40 (9.37%)
Stage IV	37 (8.67%)	9 (2.11%)	46 (10.77%)
Stage IVA	14 (3.28%)	3 (0.70%)	17 (3.98%)
Stage IVB	1 (0.23%)	1 (0.23%)	2 (0.47%)
Histological type			
Colon Adenocarcinoma	291 (68.15%)	78 (18.27%)	369 (86.42%)
Colon Mucinous Adenocarcinoma	45 (10.54%)	13 (3.04%)	58 (13.58%)

Additionally, 12 tumor samples and 12 adjacent normal samples scRNA data from GSE166555 were included, the clinicopathological characteristics of the patients are summarized (Supplementary Table S1).

### 3.2 Prognostic significance analysis of clinic features

**FIGURE 1:**
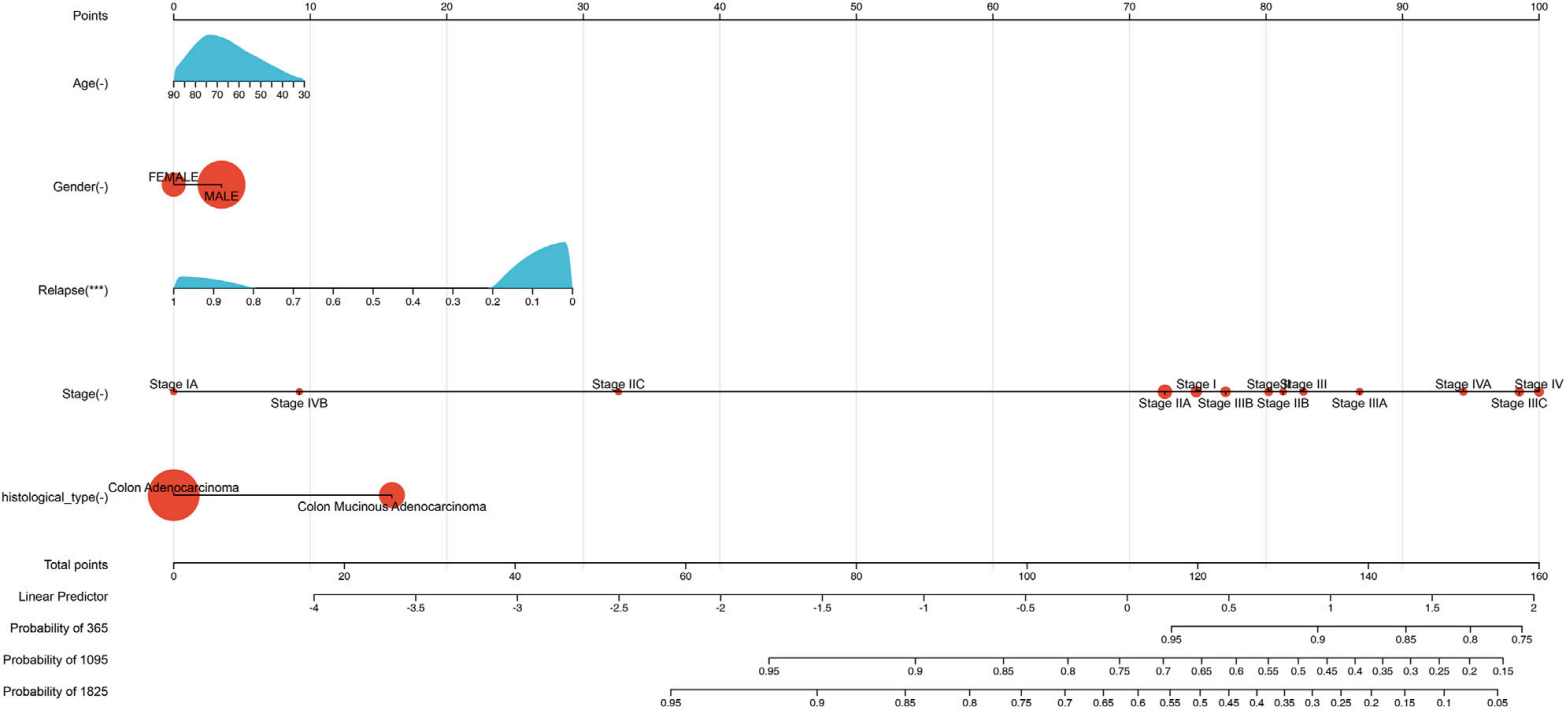
Nomogram predicting patient survival based on clinic features levels in COAD. The nomogram incorporates clinical variables such as age, gender, relapse information, histological type, and stage. Points are assigned to each variable based on their relative contribution to patient survival. The total score obtained from adding up the points for each variable can be used to estimate patient survival probability. The calibration curve shows the agreement between the predicted and observed survival probability.

We evaluated the prognostic significance of these five features in the 427 samples using the nomogram (Figure 1). The nomogram allowed us to visualize the contributions of the individual features to the overall survival outcome, and to identify which features were most strongly associated with survival.

The Cox proportional hazards regression model demonstrated a C-index of 0.687892331642646, with a 95% confidence interval of 0.62702802141112–0.748756641874171 and a statistically significant *p*-value of 1.44357293340874e-09. These findings indicate that the model has a level of predictive power above that of random prediction.

However, caution must be exercised in interpreting the clinical utility of the model. Notably, patients with higher cancer stage or relapse status may exhibit lower risk scores, indicating a potentially counterintuitive relationship between the model’s predictions and the expected clinical outcomes. Therefore, further validation studies and clinical testing are needed to assess the predictive performance of the model and determine its usefulness in clinical decision-making.

### 3.3 Differential gene expression analysis and differential immune infiltration

**FIGURE 2:**
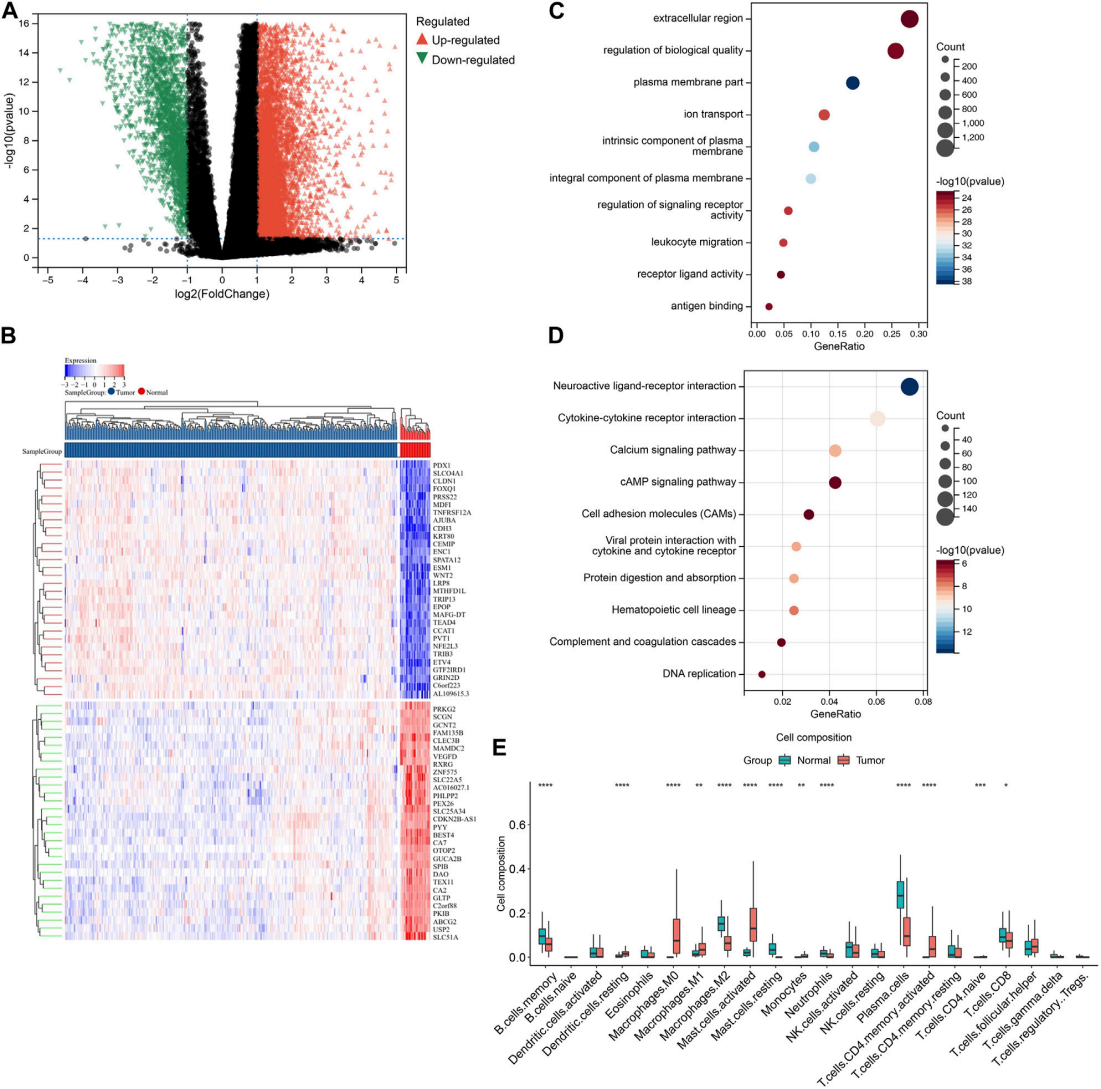
Identification and annotation of DEGs. **(A)** Volcano plot showing differential gene expression analysis between COAD and normal samples. The *x*-axis represents the log2 fold change between the two groups, and the *y*-axis represents the negative log10 of the *p*-value. The red points represent upregulated genes in COAD compared to normal samples, while the blue points represent downregulated genes. The horizontal dashed line represents the significance threshold (*p*-value <0.05), and the vertical dashed lines represent the fold change threshold (|log2FC| > 1). Genes with a significant differential expression and a fold change above the threshold are labeled. **(B)** Heatmap depicting the expression levels of DEGs between the groups. The rows represent individual genes, while the columns represent different samples. The color key shows the level of expression of the genes, with red indicating upregulation and blue indicating downregulation relative to the median expression level. The hierarchical clustering of the samples and genes is shown on the left and top of the heatmap, respectively. **(C)** GO enrichment analysis of DEGs identified in COAD samples. The color intensity of each square represents the enrichment significance, with red indicating high significance and blue indicating low significance. The size of the square reflects the number of genes associated with the GO term. **(D)** KEGG pathway analysis of DEGs identified in COAD samples. The color intensity of each square represents the enrichment significance, with red indicating high significance and blue indicating low significance. The size of the square reflects the number of genes associated with the KEGG term. **(E)** Box plot showing the relative abundance of immune cell types estimated by CIBERSORT for DEGs. The *y*-axis represents the proportion of immune cells, while the *x*-axis represents different sample groups.

To identify differentially expressed genes (DEGs) between TCGA-COAD and normal samples, we performed differential gene expression analysis using the limma package. A total of 11,583 genes were found to be differentially expressed, with 8,191 genes upregulated and 3,392 genes downregulated in TCGA-COAD samples compared to normal samples (adjusted *p*-value <0.05 and |log2 fold change| > 1). The volcano plot and heatmap of DEGs is shown in Figures 2A, B.

To investigate the biological functions and pathways associated with these differentially expressed genes, we performed gene ontology (GO) and KEGG pathway analysis (Figures 2C, D). Although these functions may not be directly implicated in immune infiltration, they are strongly linked to this process. Consequently, we employed CIBERSORT to conduct further investigation of the differential immune infiltration (Figure 2E).

**FIGURE 3:**
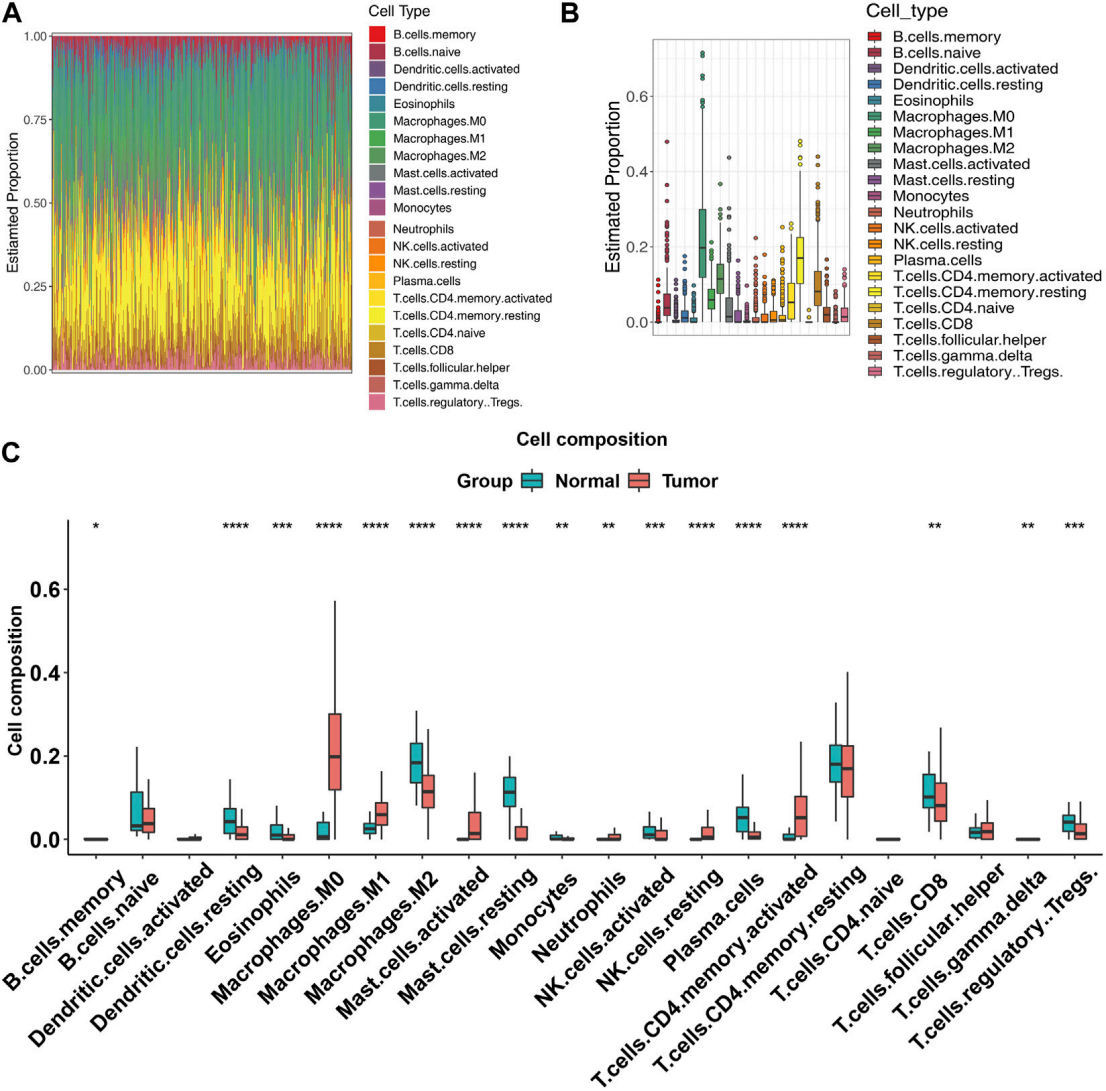
Immune infiltration of tumor samples. **(A)** Bar plot showing the expression levels of all the genes in COAD patients. Each bar represents the mean expression level of a gene in tumor samples. **(B)** Box plot showing the distribution of expression levels of all the genes in COAD patients. The box represents the interquartile range (IQR), with the median (horizontal line), and the whiskers indicate the range of values within 1.5 times the IQR from the upper and lower quartiles. Outliers are shown as individual points. **(C)** Box plot showing the distribution of expression levels of all the genes in COAD patients and normal samples, each box represents the proportions of an immune cell type in tumor samples (red) and normal samples (blue).

In view of the significant differences observed in immune infiltration associated with the differentially expressed genes, we performed CIBERSORT again across all expression profiles to further elucidate this relationship. The bar plot and box plot revealed substantial immune heterogeneity in the tumor samples (Figures 3A, B). The immune infiltration analysis revealed a significant difference in the composition of immune cells between tumor and normal tissues. In tumor samples, there was a decrease in the abundance of DC resting, eosinophils, macrophages M1, macrophages M2, mast cells resting, monocytes, NK cells activated, plasma cells, CD8^+^ T cells, and Tregs (*p* < 0.05) compared to normal samples. On the other hand, macrophages M0, NK cells resting, and CD4^+^ memory activated were found to be significantly more abundant in tumor samples (*p* < 0.05). These findings suggest a potential role for these immune cell subsets in the development and progression of the tumor (Figure 3C).

### 3.4 The impact of distinct immune infiltration on survival

**FIGURE 4:**
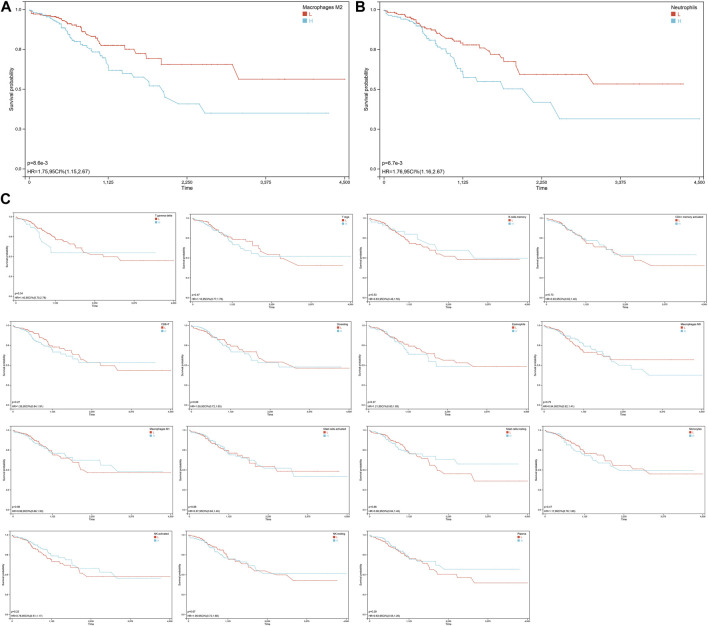
Survival analysis of COAD patients base on the different immune infiltration. **(A)** KM curve for the association of macrophage M2 infiltration and overall survival in COAD patients. The high macrophage M2 infiltration group is indicated in blue, while the low macrophage M2 infiltration group is indicated in red. The X-axis represents the survival time in days, and the Y-axis represents the survival probability. The *p*-value was calculated using the log-rank test. The KM curve suggests that COAD patients with high macrophage M2 infiltration have a significantly worse prognosis than those with low macrophage M2 infiltration. **(B)** KM curve for the association of neutrophils infiltration and overall survival in COAD patients. **(C)** KM curves of immune cells with significant differences but no significant impact on survival.

To assess the impact of different immune cell infiltration on COAD patients’ survival, we divided the COAD samples into two groups based on the median proportion of different abundance immune cell type. Our analysis revealed that the survival of patients was significantly impacted by the proportion of macrophages M2 (HR = 1.75, Figure 4A) and neutrophils (HR = 1.76, Figure 4B). Higher proportions of macrophages M2 or neutrophils were associated with a worse prognosis, the proportions of other immune cells did not have a significant impact on patient survival (Figure 4C).

Next, we further investigated the genes related to neutrophils and macrophages. Due to the limitations in the annotation of single-cell RNA sequencing data, we could not specifically focus on macrophages M2 as the subtype could not be precisely divided.

**FIGURE 5:**
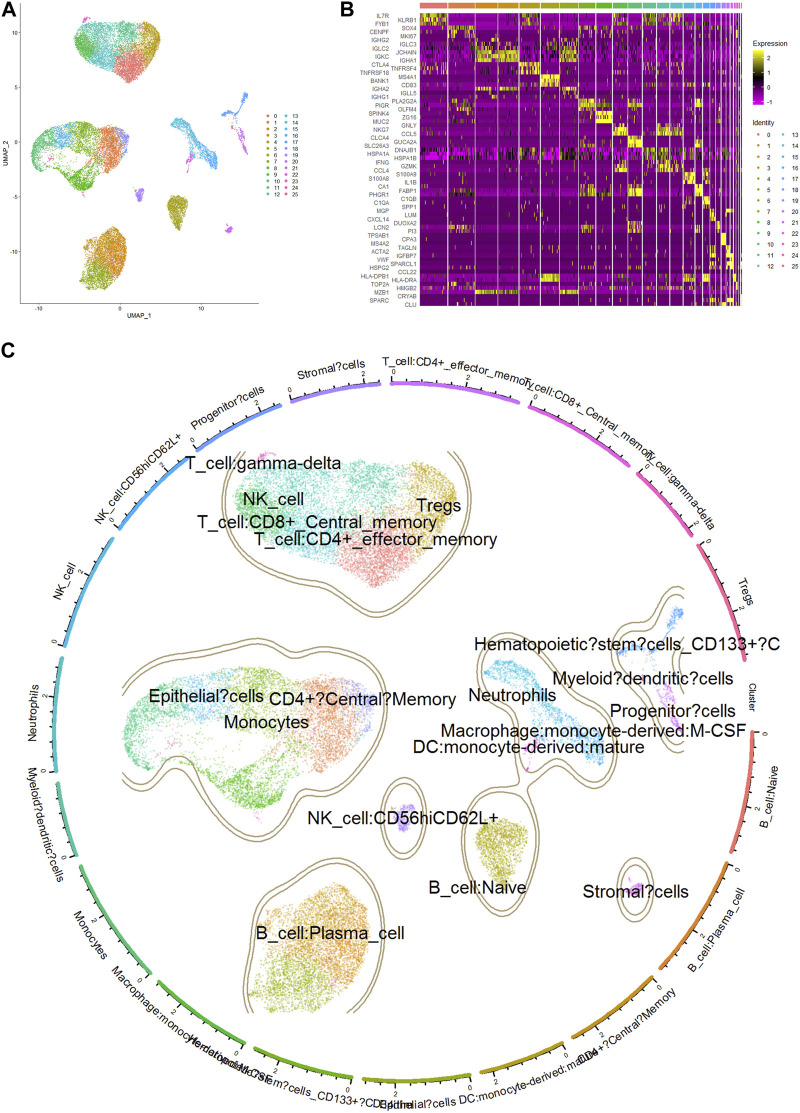
Cell clusters, markers and annotation of scRNA sequencing data. **(A)** UMAP plot of single-cell gene expression data colored by cell type. Cell types were identified using the Seurat package based on DEGs. **(B)** Heatmap showing the expression of top 3 marker genes in single cells of COAD. Rows represent individual cell clusters, and columns represent the expression levels of marker genes. The heatmap is color-coded, with yellow indicating high expression and purple indicating low expression. The top 3 marker genes are labeled on the left side of the heatmap. **(C)** Annotation of clusters by marker genes highly expressed in certain cell types.

To obtain the genes representative of these immune cells, we analyzed the single-cell RNA sequencing data of COAD samples using the Seurat package. The differentially expressed genes in CD8^+^ T cells and dendritic cells were identified using the FindAllMarkers function of Seurat package. The gene expression profiles of these cells were compared to that of other cell types to ensure their specificity. A total of 26 cell clusters were identified and annotated based on known cell type markers and functional annotations (Figures 5A, B). Cluster 14 was identified as neutrophils (markers: S100A9, S100A8, IL1B), cluster 16 as macrophages (markers:C1QB, C1QA, and SPP1) (Figure 5C). The distribution of cell types varied between tumor and normal samples, indicating a significant change in the cellular composition of the tumor microenvironment. After filtering the differentially expressed genes, a total of 898 genes representing neutrophils and 1,088 genes representing macrophages were selected for subsequent analysis.

### 3.5 Predict model base on genes related to neutrophils and macrophages

A new matrix containing 898 genes representing neutrophils and 1,088 genes representing macrophages was created for subsequent univariate cox regression analysis.

Univariate Cox regression analysis was performed to evaluate the association between each gene representing neutrophils or macrophages and patient survival. 365 genes that were found to be significantly associated with survival in univariate analysis were then included in the lasso Cox regression analysis.

**FIGURE 6:**
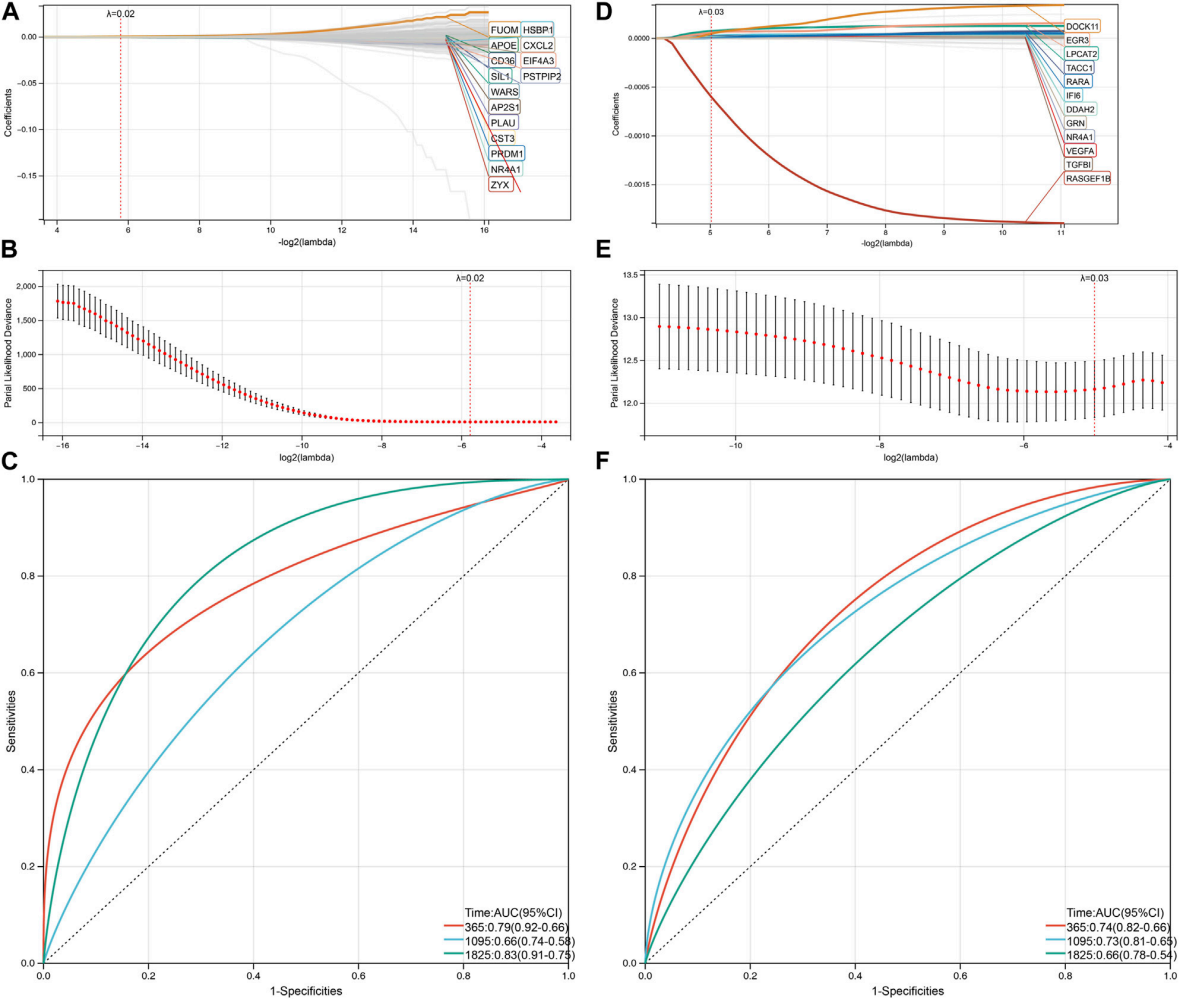
Predict model based on genes related to neutrophils and macrophages. **(A)** The lasso cox model was used to identify the optimal prognostic gene signature among a large number of genes. The *y*-axis represents the coefficients of the selected factors, and the *x*-axis represents the -log10 (lambda) of the factors. **(B)** The selection of the optimal lambda value in a lasso cox model was performed using 10-fold cross-validation. The lambda value that produced the minimum partial likelihood deviance was chosen as the optimal lambda value. **(C)** ROC analysis was performed to evaluate the predictive ability of the lasso cox model. The AUC value and 95% confidence interval (CI) are shown in the figure. **(D)** The lasso cox model of the optimal prognostic gene signature relates to COAD relapse. **(E)** The selection of the optimal lambda value in relapse lasso cox model. **(F)** ROC analysis was performed to evaluate the predictive ability of the relapse lasso cox model.

To further refine the prognostic factors, the lasso Cox regression analysis was used to identify the most important genes that are associated with patient survival. The final model was determined based on the optimal tuning parameter and cross-validation error. The resulting model incorporated 16 genes, with a Lambda value of 0.0180964575552417 (Figures 6A, B).

$$\begin{array}{ll} RiskScore & =-9.27892150816884e-05*HSBP1-7.39529848766097e-05*SIL1+0.000232190435630512*CD36\\ & -3.30705339703081e-05*EIF4A3-9.62564217960586e-06*PSTPIP2-6.68757505192394e\\ & -06*NR4A1-2.21789565393933e-05*HIF1A-2.79680024122654e-05*AP2S1-4.45418222479386e\\ & -05*CXCL2-1.6363573605246e-05*ZYX-0.000128852882734705*PRDM1\\ & +0.0005791148853528*FUOM+1.74412406364351e-05*APOE-4.42195522736364e-05*PLAU\\ & +1.2356458827137e-05*WARS-3.44575724547677e-06*CST3 \end{array}$$

To evaluate the predictive performance of the model, receiver operating characteristic (ROC) analyses were conducted for three different time points: 365 days, 1,095 days, and 1,825 days. The resulting AUC values were 0.79, 0.66, and 0.83, respectively (Figure 6C).

These findings suggest that the 16-gene model derived from lasso Cox regression, based on genes selected using univariate Cox regression analysis, exhibits potential as a prognostic tool for predicting patient outcomes.

We repeated the aforementioned analysis using relapse information from COAD patients. After conducting a univariate Cox regression analysis with a logrank threshold of less than 0.05, 26 genes were selected for further analysis with lasso Cox regression. The model obtained from the analysis consisted of 12 genes, with a corresponding Lambda value of 0.0308784474015521 (Figures 6D, E).

$$\begin{array}{ll} RiskScore & =2.79395070106422e-05*IFI6+7.5275421600356e-05*LPCAT2+1.97276329697907e\\ & -05*TACC1+5.81813600394489e-05*EGR3+7.06009460176273e\\ & -06*NR4A1+1.41114162602005e-05*DDAH2+3.91852689977715e\\ & -06*VEGFA+1.32167767127812e-06*TGFBI+1.21367456488834e\\ & -06*GRN-0.000600466175445024*RASGEF1B+2.82592973016063e\\ & -06*RARA+4.73122291984313e-05*DOCK11 \end{array}$$

ROC analysis of the data yielded the following results: AUC of 0.74 for 365 days, 0.73 for 1,095 days, and 0.66 for 1,825 days (Figure 6F).

**FIGURE 7:**
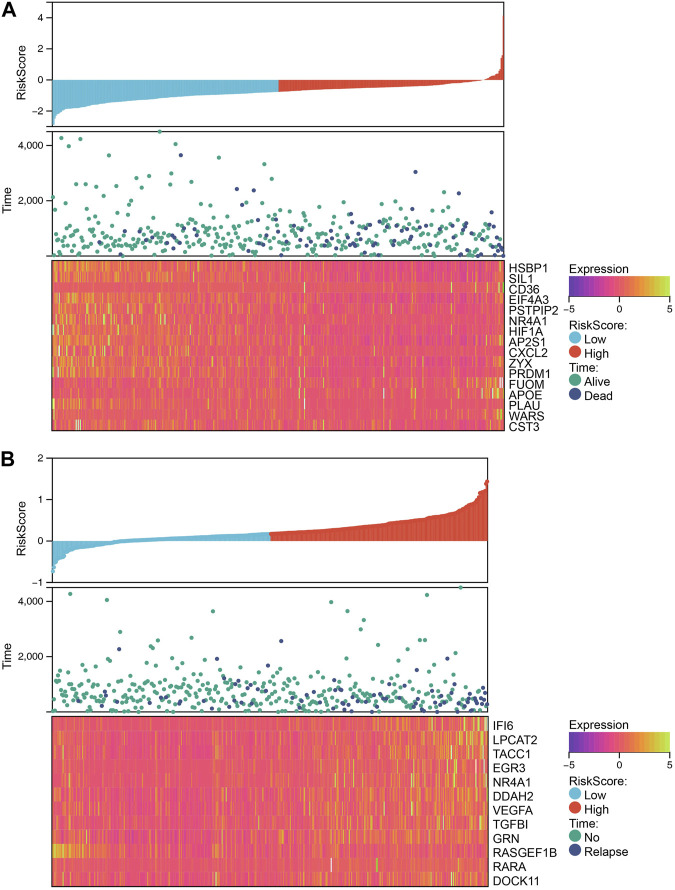
The relationship between different risk scores and patients’ follow-up time, events, and expression changes of various genes. **(A)** The relationship between different risk scores and overall survival time, living events, and expression changes of model genes. **(B)** The relationship between different risk scores and relapse time, relapse events, and expression changes of model genes.

Our analysis demonstrated that patients with higher RiskScore had a poorer prognosis, indicating the potential utility of this metric as a prognostic tool. The calculation of RiskScore was based on the expression levels of genes, with those exhibiting a positive coefficient indicating an unfavorable prognosis and those with a negative coefficient indicating a protective effect (Figures 7A, B). The identification of NR4A1 that was present in both models suggests that it may be a key factor in determining prognosis.

In order to validate the performance of our model, we performed an external validation using two independent data sets. We calculated the risk scores using the same formula as the training dataset, and generated ROC curves to evaluate the predictive performance of the model. The resulting AUC values for both data sets were greater than 0.5, indicating that the model had some predictive ability (Supplementary Figure S2). However, we observed that the performance of the model was not as strong as the training dataset. This could be due to the fact that some genes included in the training signature may not have been detected or measured in the external validation datasets due to differences in the sequencing chips used or other technical variations.

### 3.6 Chemical compounds sensitivities

**FIGURE 8:**
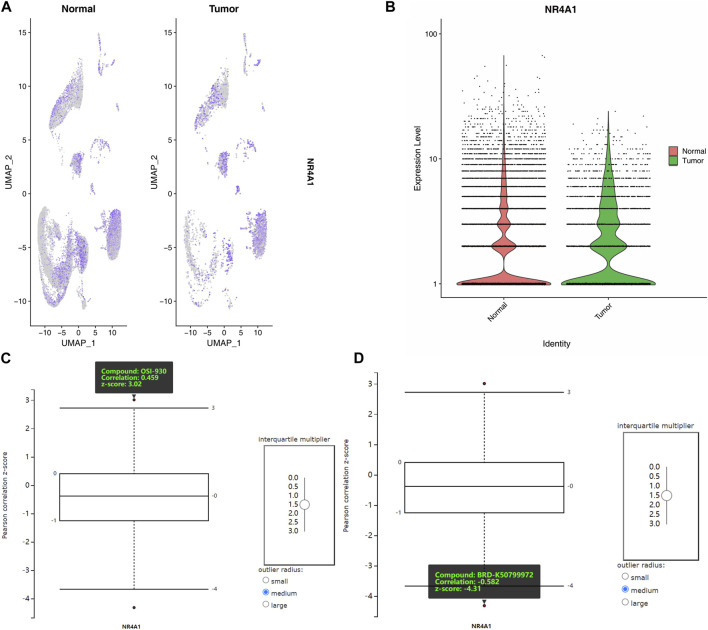
Expression, distribution and drug sensitivities analysis of NR4A1. **(A)** The gene expression levels of NR4A1 in different cell clusters. **(B)** The total expression of NR4A1 in COAD and normal samples scRNA data. **(C)** The positive correlation of NR4A1 expression and OSI-930. **(D)** The negative correlation of NR4A1 expression and BRD-K50799972.

To further evaluate the involvement of NR4A1 as a pivotal factor, we conducted a single-cell RNA sequencing analysis to assess its expression levels and distribution. Our findings revealed that the distribution of NR4A1 was more extensive, and the expression levels were higher in tumor samples as compared to normal colon samples (Figures 8A, B). We obtained protein staining results for NR4A1 from the Human Protein Atlas for analysis. The results showed that the staining intensity of NR4A1 was stronger in COAD samples compared to normal colon samples (Supplementary Figure S3). These finding suggests that NR4A1 may play a role in the development and progression of colon cancer.

To analyze the correlation between NR4A1 expression and chemical compound sensitivities, we used the Cancer Therapeutics Response Portal (CTRP) v2 database. This database contains gene expression data from various cancer cell lines and their responses to chemical compounds.

To focus on the large intestine, a site-specific filter was applied, and an interquartile multiplier cutoff of 1.5 was used to identify differentially expressed genes. Subsequently, the correlation between the expression levels of NR4A1 and two compounds, OSI-930 and BRD-K50799972, was examined. The analysis revealed a positive correlation between OSI-930 and NR4A1 expression (Correlation: 0459, z-score: 3.02, Figure 8C), while BRD-K50799972 showed a negative correlation with NR4A1 expression (Correlation: −0.582, z-score: −4.31, Figure 8D).

The analysis suggests that OSI-930 and BRD-K50799972 have different sensitivities for the NR4A1 expression. This information could be useful in the development of targeted therapies for diseases that involve dysregulation of the NR4A1. For example, OSI-930 may be a potential candidate for patients who have higher sensitivity to the compound, while BRD-K50799972 may be useful for patients who have lower sensitivity to the compound. However, further studies are needed to confirm the efficacy and safety of these compounds in treating colon adenocarcinoma.

## 4 Discussion

COAD is a common malignant tumor that has a high morbidity and mortality rate worldwide (Siegel et al., 2021). The complexity and heterogeneity of CRC have made it challenging to identify specific therapeutic targets, and there is an urgent need to develop new approaches to combat this disease. Recently, single-cell sequencing has emerged as a powerful tool for investigating the genetic and molecular mechanisms underlying cancer pathogenesis (Wei et al., 2023). In this study, we used single cell sequencing to identify key genes and pathways associated with COAD. Our approach allowed us to analyze the transcriptional profiles of individual cells, enabling us to identify cell type-specific changes in gene expression that would have been masked in bulk sequencing analysis. By doing so, we aimed to improve our understanding of the disease and identify potential therapeutic targets that could help to improve patient outcomes.

In this study, we investigated various clinic features (Age, Gender, Stage, Relapse, and Histological type) and their association with patient outcomes. Surprisingly, our analysis did not reveal any significant direct link between clinic features and patient prognosis. These findings suggest that other factors, such as molecular alterations or immune cell infiltration, may have a more significant impact on patient outcomes in this particular cancer type.

The differential expression analysis identified a total of 11,583 genes that were significantly dysregulated between COAD and normal samples. These differentially expressed genes are likely to contribute to the differences in immune infiltration observed between the two groups (As Figure 3 shown). Functional analysis of differentially expressed genes (DEGs) revealed multiple immune-associated pathways that can impact various aspects of immune function and contribute to immune infiltration in different cancers. The Neuroactive ligand-receptor interaction, cytokine-cytokine receptor interaction, Calcium signaling pathway, Viral protein interaction with cytokine and cytokine receptor, Protein digestion and absorption, Hematopoietic cell lineage, DNA replication, Complement and coagulation cascades, cAMP signaling pathway, and Cell adhesion molecules (CAMs) pathways were found to be associated with immune infiltration. These pathways are involved in immune cell migration, modulation of the inflammatory response, immune cell proliferation, differentiation, activation, T-cell activation, cytokine production, chemotaxis, immune response to viral infection, macrophage infiltration, development and differentiation of immune cells, recruitment of immune cells to the site of inflammation, and regulation of leukocyte trafficking and recruitment to the site of inflammation (Su et al., 2003; Ota et al., 2007).

Hence, we focused on the immune heterogeneity between COAD samples. We found that different immune infiltrates in COAD can have varying impacts on patient prognosis. Specifically, patients with a higher proportion of macrophages M2 or neutrophils are associated with worse outcomes. To better understand the molecular mechanisms underlying this association, we focused on genes from clusters related to macrophages M2 or neutrophils. Using these genes, we created two predictive models that can help identify patients who are at a higher risk of poor outcomes based on their immune infiltration patterns. These models have the potential to be used clinically to guide treatment decisions and improve patient outcomes. The findings of our study highlight the potential of NR4A1 as a therapeutic target in COAD. The observed protective effect of NR4A1 in both models suggests its role in regulating tumor survival and relapse. Cancer therapeutics response analysis revealed that the expression level of NR4A1 can influence the response to specific drugs. High expression levels of NR4A1 may benefit from treatment with OSI-930, while those with low expression levels may respond better to BRD-K50799972. These results suggest that targeting NR4A1 expression may be a promising approach to improve cancer treatment outcomes.

NR4A1, also known as Nur77, is a transcription factor that has been implicated in the development and progression of various types of cancer, including COAD. Studies have shown that NR4A1 plays a crucial role in the development and progression of various diseases, such as cancer, cardiovascular disease, metabolic disorders, and neurodegenerative diseases. NR4A1 is also involved in the modulation of cellular signaling pathways, such as the PI3K/AKT/mTOR, MAPK/ERK, and Wnt/β-catenin pathways, through its transcriptional activity or protein-protein interactions (Li et al., 2018; Zeng et al., 2018). Furthermore, the absence or loss of NR4A1 expression may result in an impaired ability to differentiate into M2 macrophages (Murphy et al., 2014).

OSI-930 is a potent and selective inhibitor of c-Kit, a receptor tyrosine kinase that is overexpressed in a variety of cancers, including gastrointestinal stromal tumors (GISTs) and acute myeloid leukemia (AML). OSI-930 has shown promising results in preclinical studies and has entered clinical trials as a potential anticancer agent (Malaise et al., 2009). *In vitro* and *in vivo* studies have demonstrated that OSI-930 can inhibit c-Kit activity and downstream signaling pathways, leading to inhibition of cancer cell proliferation and induction of apoptosis (Garton et al., 2006). Additionally, OSI-930 has been shown to have synergistic effects with other anticancer agents, such as imatinib, in the treatment of c-Kit-positive tumors (Malaise et al., 2009). Therefore, targeting NR4A1 may be a potential therapeutic strategy for COAD treatment. Although some studies have investigated the therapeutic potential of BRD-K50799972, there is currently limited research available on its efficacy and safety, and it has not yet progressed to the preclinical stage. Therefore, further investigation is needed to determine its potential as a therapeutic agent. For the purpose of this study, we have not included BRD-K50799972 in our analysis or discussion.

In conclusion, our study analyzed the differential gene expression profiles and immune infiltration in COAD samples. We identified several differentially expressed genes and pathways associated with immune processes in COAD, which may contribute to immune infiltration and cancer progression. Specifically, we found that high expression levels of NR4A1 were associated with a better prognosis in COAD patients. We also identified OSI-930 as a potential therapeutic agent for COAD patients with high NR4A1 expression levels. Our study provides insights into the molecular mechanisms underlying COAD and identifies potential therapeutic targets for this disease. However, further experimental validation is required to confirm our findings.

## Data Availability

The original contributions presented in the study are included in the article/Supplementary Material, further inquiries can be directed to the corresponding author.
