# Supplementary material for: NR4A1 as a potential therapeutic target in colon adenocarcinoma: a computational analysis of immune infiltration and drug response
Source: Front Genet. 2023 Jul 26;14:1181320. doi: 10.3389/fgene.2023.1181320 (PMC10410285; doi:10.3389/fgene.2023.1181320)
Supplement: Supplementary file 1 [file Table1.DOCX]

| Characteristics | Total(N=12) |
| --- | --- |
| T |  |
| Tis | 1(8.3%) |
| T1 | 1(8.3%) |
| T2 | 4(33.3%) |
| T3 | 4(33.3%) |
| T4 | 2(16.7%) |
| N |  |
| N0 | 6(50%) |
| N1 | 3(25%) |
| N2 | 3(25%) |
| M |  |
| M0 | 11(91.7%) |
| M1 | 1(8.3%) |
| Grade |  |
| G2 | 8(66.7%) |
| G3 | 4(33.3%) |
| Immunohistology |  |
| MSS | 10(83.3%) |
| MSI | 1(8.3%) |
| N/A | 1(8.3%) |

**Supplementary table 1. The clinicopathological characteristics of 12 enrolled scRNA sequencing patients.**

| Tag | Time | Status | RiskScore |
| --- | --- | --- | --- |
| TCGA-AA-3979-01 | 730 | 0 | -0.20368 |
| TCGA-AA-3655-01 | 1856 | 0 | -1.34539 |
| TCGA-D5-6535-01 | 460 | 1 | -0.82043 |
| TCGA-CK-4950-01 | 2599 | 0 | -1.72691 |
| TCGA-A6-3810-01 | 1111 | 0 | -0.76857 |
| TCGA-AD-6888-01 | 472 | 1 | -1.14281 |
| TCGA-AA-3680-01 | 335 | 0 | -0.24381 |
| TCGA-3L-AA1B-01 | 475 | 0 | -1.2137 |
| TCGA-AY-4070-01 | 496 | 0 | -0.50043 |
| TCGA-CM-6675-01 | 397 | 0 | -0.94081 |
| TCGA-A6-6650-01 | 627 | 0 | -0.93458 |
| TCGA-D5-6898-01 | 229 | 0 | -0.74681 |
| TCGA-QG-A5Z1-01 | 256 | 0 | -1.33057 |
| TCGA-AA-A00J-01 | 549 | 0 | -0.57753 |
| TCGA-A6-6138-01 | 685 | 0 | -0.95092 |
| TCGA-NH-A8F8-01 | 511 | 0 | -0.43653 |
| TCGA-CK-6751-01 | 518 | 1 | -0.74289 |
| TCGA-D5-6534-01 | 1316 | 0 | -0.47962 |
| TCGA-A6-2677-01 | 740 | 0 | -0.74356 |
| TCGA-T9-A92H-01 | 362 | 0 | -1.43354 |
| TCGA-AA-3506-01 | 1765 | 0 | -0.246 |
| TCGA-AD-6890-01 | 746 | 1 | -0.80553 |
| TCGA-DM-A1D4-01 | 2821 | 0 | -1.30737 |
| TCGA-AA-A00F-01 | 1035 | 0 | -0.51483 |
| TCGA-A6-6780-01 | 612 | 1 | 0.004458 |
| TCGA-AA-3939-01 | 395 | 0 | -0.98884 |
| TCGA-AA-3679-01 | 457 | 1 | -0.49881 |
| TCGA-DM-A285-01 | 179 | 0 | -1.83543 |
| TCGA-NH-A6GB-01 | 476 | 0 | -1.27793 |
| TCGA-AA-3972-01 | 1551 | 0 | -0.66486 |
| TCGA-A6-2675-01 | 1321 | 1 | -0.77238 |
| TCGA-G4-6321-01 | 672 | 1 | -0.20895 |
| TCGA-CK-5916-01 | 643 | 0 | -0.8065 |
| TCGA-DM-A28M-01 | 2895 | 0 | -1.25691 |
| TCGA-CM-5344-01 | 670 | 1 | -1.23623 |
| TCGA-AA-3561-01 | 424 | 0 | -0.92303 |
| TCGA-AD-6965-01 | 805 | 0 | -0.62808 |
| TCGA-QG-A5YV-01 | 1301 | 1 | -0.82049 |
| TCGA-A6-5656-01 | 1001 | 0 | -0.97323 |
| TCGA-AD-6548-01 | 650 | 0 | -0.77684 |
| TCGA-A6-6651-01 | 662 | 0 | -1.16063 |
| TCGA-CM-6166-01 | 669 | 0 | -0.89601 |
| TCGA-CM-6162-01 | 365 | 0 | -1.95368 |
| TCGA-AA-3870-01 | 912 | 1 | -0.58964 |
| TCGA-AA-A00R-01 | 30 | 0 | -0.14125 |
| TCGA-D5-5537-01 | 1381 | 0 | -1.23472 |
| TCGA-AA-3509-01 | 1915 | 0 | -0.80999 |
| TCGA-AA-3815-01 | 1005 | 0 | -0.41244 |
| TCGA-F4-6806-01 | 1260 | 0 | -0.85111 |
| TCGA-A6-6142-01 | 763 | 0 | -1.21751 |
| TCGA-AA-3544-01 | 426 | 0 | -0.20352 |
| TCGA-DM-A1DB-01 | 1348 | 0 | -1.67046 |
| TCGA-A6-2678-01 | 1286 | 0 | -0.66274 |
| TCGA-AA-3664-01 | 1643 | 0 | -0.36034 |
| TCGA-AA-3660-01 | 2375 | 1 | -0.83467 |
| TCGA-AA-3666-01 | 61 | 0 | -0.51903 |
| TCGA-CM-6678-01 | 335 | 0 | -1.67452 |
| TCGA-A6-2672-01 | 1419 | 0 | -0.58155 |
| TCGA-CM-5868-01 | 518 | 0 | -0.99396 |
| TCGA-A6-2686-01 | 1126 | 0 | 0.068744 |
| TCGA-A6-2682-01 | 424 | 0 | -0.44741 |
| TCGA-G4-6304-01 | 1631 | 0 | -0.30114 |
| TCGA-CK-5914-01 | 669 | 0 | -1.75709 |
| TCGA-D5-5541-01 | 1701 | 1 | -0.41516 |
| TCGA-AA-A00U-01 | 518 | 0 | -0.56164 |
| TCGA-AA-3532-01 | 882 | 1 | -0.76512 |
| TCGA-DM-A1D8-01 | 383 | 0 | -0.6075 |
| TCGA-AM-5820-01 | 14 | 0 | -0.09475 |
| TCGA-F4-6855-01 | 1442 | 0 | -0.79393 |
| TCGA-A6-6781-01 | 598 | 0 | -2.18126 |
| TCGA-CA-6716-01 | 371 | 0 | -1.34041 |
| TCGA-D5-6927-01 | 287 | 0 | -0.79102 |
| TCGA-A6-6653-01 | 742 | 0 | -1.38101 |
| TCGA-AA-3864-01 | 1612 | 0 | -0.72545 |
| TCGA-DM-A1DA-01 | 228 | 1 | 1.587851 |
| TCGA-F4-6704-01 | 47 | 0 | -1.80565 |
| TCGA-AA-3552-01 | 396 | 0 | -0.38822 |
| TCGA-AA-A02H-01 | 61 | 1 | -0.19231 |
| TCGA-DM-A28K-01 | 2988 | 0 | -1.10726 |
| TCGA-AZ-4323-01 | 43 | 0 | -1.22717 |
| TCGA-A6-A565-01 | 494 | 0 | -1.07588 |
| TCGA-F4-6459-01 | 262 | 0 | -0.87819 |
| TCGA-G4-6317-01 | 1095 | 1 | -0.45467 |
| TCGA-AA-3520-01 | 731 | 0 | -0.70092 |
| TCGA-AA-3941-01 | 730 | 0 | -0.0535 |
| TCGA-F4-6569-01 | 1087 | 0 | -0.89345 |
| TCGA-D5-6926-01 | 275 | 1 | -1.1038 |
| TCGA-G4-6297-01 | 2506 | 0 | -1.58468 |
| TCGA-QG-A5YX-01 | 1003 | 1 | -1.05256 |
| TCGA-A6-A5ZU-01 | 293 | 0 | -0.25695 |
| TCGA-AY-A69D-01 | 543 | 0 | -1.96929 |
| TCGA-A6-6649-01 | 735 | 0 | -1.13451 |
| TCGA-CK-5913-01 | 1561 | 0 | -1.41908 |
| TCGA-4N-A93T-01 | 146 | 0 | -1.05021 |
| TCGA-AA-3821-01 | 31 | 0 | -0.38913 |
| TCGA-AA-3970-01 | 1096 | 1 | -0.3829 |
| TCGA-AA-3525-01 | 1 | 0 | -0.34183 |
| TCGA-CM-6167-01 | 456 | 0 | -0.46093 |
| TCGA-A6-5661-01 | 1020 | 0 | -0.96782 |
| TCGA-AA-A01P-01 | 1158 | 0 | 0.046457 |
| TCGA-D5-6539-01 | 380 | 0 | -0.61668 |
| TCGA-AA-3818-01 | 30 | 0 | -0.83274 |
| TCGA-AA-3681-01 | 182 | 0 | -0.37888 |
| TCGA-G4-6625-01 | 2792 | 0 | -0.79017 |
| TCGA-AA-3514-01 | 31 | 0 | -0.62929 |
| TCGA-F4-6809-01 | 403 | 1 | 0.581333 |
| TCGA-AA-3966-01 | 61 | 0 | -0.49898 |
| TCGA-AA-A00L-01 | 1157 | 0 | -0.42286 |
| TCGA-AA-3950-01 | 730 | 0 | -0.49849 |
| TCGA-AA-3866-01 | 518 | 0 | -0.57712 |
| TCGA-CM-6674-01 | 394 | 0 | -0.53057 |
| TCGA-A6-2676-01 | 1305 | 0 | -0.20004 |
| TCGA-AA-3548-01 | 1034 | 0 | -0.57599 |
| TCGA-CK-4948-01 | 4502 | 0 | -1.17364 |
| TCGA-G4-6627-01 | 2175 | 0 | -1.5161 |
| TCGA-AA-3844-01 | 454 | 0 | -0.8161 |
| TCGA-A6-5657-01 | 962 | 0 | -1.60309 |
| TCGA-AU-6004-01 | 824 | 0 | -0.89849 |
| TCGA-D5-6922-01 | 308 | 0 | -1.27466 |
| TCGA-AA-3662-01 | 184 | 1 | -0.86095 |
| TCGA-G4-6307-01 | 1674 | 0 | -2.47907 |
| TCGA-A6-6648-01 | 766 | 0 | -1.81574 |
| TCGA-A6-2674-01 | 1331 | 0 | -1.02516 |
| TCGA-CM-6168-01 | 395 | 0 | -1.32152 |
| TCGA-CM-4752-01 | 396 | 0 | -0.59387 |
| TCGA-DM-A1D0-01 | 3974 | 0 | -1.84786 |
| TCGA-AA-A00Z-01 | 669 | 0 | -0.6252 |
| TCGA-CM-4743-01 | 701 | 0 | -1.0459 |
| TCGA-AA-3930-01 | 61 | 1 | -0.03408 |
| TCGA-AA-A02O-01 | 28 | 1 | -0.75452 |
| TCGA-AA-3831-01 | 547 | 0 | -0.42928 |
| TCGA-A6-5659-01 | 926 | 0 | -1.80612 |
| TCGA-AA-3973-01 | 397 | 1 | -0.55263 |
| TCGA-CM-4744-01 | 609 | 0 | 0.692617 |
| TCGA-D5-6929-01 | 408 | 1 | -0.80809 |
| TCGA-AA-3982-01 | 822 | 0 | -0.32837 |
| TCGA-AA-3554-01 | 546 | 1 | -0.59766 |
| TCGA-AZ-4616-01 | 156 | 0 | -0.93926 |
| TCGA-AA-3956-01 | 1035 | 1 | -0.38951 |
| TCGA-D5-6931-01 | 365 | 0 | -0.91414 |
| TCGA-AA-A01D-01 | 334 | 0 | -0.38394 |
| TCGA-AA-3986-01 | 580 | 0 | -0.53239 |
| TCGA-DM-A28F-01 | 1094 | 0 | -1.84614 |
| TCGA-DM-A0X9-01 | 3641 | 0 | -1.4629 |
| TCGA-AA-3837-01 | 1186 | 1 | -0.14732 |
| TCGA-AA-3495-01 | 1127 | 0 | -0.46894 |
| TCGA-F4-6703-01 | 1456 | 0 | -0.66414 |
| TCGA-AA-A00D-01 | 578 | 0 | 0.014519 |
| TCGA-AA-3685-01 | 1127 | 0 | -1.07677 |
| TCGA-AZ-4615-01 | 1002 | 0 | -0.26175 |
| TCGA-NH-A8F7-01 | 543 | 0 | -0.91252 |
| TCGA-AA-A024-01 | 1188 | 0 | -0.58222 |
| TCGA-NH-A50T-01 | 553 | 0 | -1.09701 |
| TCGA-CM-5860-01 | 974 | 0 | -1.28627 |
| TCGA-CA-5256-01 | 379 | 0 | -0.87597 |
| TCGA-CM-6680-01 | 366 | 0 | -1.39853 |
| TCGA-CA-6715-01 | 383 | 0 | -1.46781 |
| TCGA-G4-6322-01 | 792 | 0 | -2.31078 |
| TCGA-AA-3511-01 | 212 | 0 | -1.46 |
| TCGA-D5-6540-01 | 491 | 0 | -1.30034 |
| TCGA-D5-6920-01 | 377 | 0 | -1.81194 |
| TCGA-G4-6586-01 | 1089 | 0 | -0.7237 |
| TCGA-AA-A029-01 | 1581 | 1 | 0.257155 |
| TCGA-AA-3875-01 | 549 | 0 | -0.27186 |
| TCGA-CM-6677-01 | 337 | 0 | -0.69614 |
| TCGA-AA-3688-01 | 578 | 0 | -0.87445 |
| TCGA-AA-A01T-01 | 1005 | 0 | -0.69519 |
| TCGA-G4-6295-01 | 254 | 0 | -0.20826 |
| TCGA-F4-6807-01 | 1309 | 0 | -1.01734 |
| TCGA-AZ-6598-01 | 1503 | 0 | -0.55332 |
| TCGA-AA-A01G-01 | 365 | 1 | -0.53788 |
| TCGA-AA-A01F-01 | 974 | 1 | -0.31274 |
| TCGA-CM-6161-01 | 457 | 0 | -1.14863 |
| TCGA-AA-3549-01 | 639 | 0 | -0.65053 |
| TCGA-CM-6164-01 | 883 | 0 | -1.6111 |
| TCGA-D5-6537-01 | 146 | 0 | -2.15006 |
| TCGA-D5-6533-01 | 775 | 0 | -1.27752 |
| TCGA-A6-2683-01 | 504 | 0 | -0.76371 |
| TCGA-CM-4747-01 | 761 | 0 | -0.57882 |
| TCGA-AA-3949-01 | 791 | 0 | -0.08891 |
| TCGA-AZ-4682-01 | 680 | 0 | -1.91341 |
| TCGA-AD-6899-01 | 176 | 0 | -1.15915 |
| TCGA-CM-6169-01 | 396 | 1 | -0.45849 |
| TCGA-AA-3673-01 | 1522 | 1 | -0.54668 |
| TCGA-CM-5863-01 | 457 | 1 | -0.54283 |
| TCGA-AA-3980-01 | 242 | 0 | -0.63974 |
| TCGA-AZ-5407-01 | 2683 | 0 | -1.02035 |
| TCGA-D5-7000-01 | 312 | 0 | -0.92327 |
| TCGA-F4-6461-01 | 338 | 0 | -0.8875 |
| TCGA-CM-6165-01 | 488 | 0 | -1.04192 |
| TCGA-AA-3489-01 | 214 | 0 | -0.21678 |
| TCGA-AA-3517-01 | 1186 | 0 | -0.48146 |
| TCGA-DM-A282-01 | 4233 | 0 | -1.77446 |
| TCGA-D5-6530-01 | 621 | 0 | -0.85675 |
| TCGA-AA-A00O-01 | 822 | 1 | -0.11762 |
| TCGA-A6-2679-01 | 1366 | 0 | -0.42736 |
| TCGA-AA-3851-01 | 1006 | 0 | -0.80018 |
| TCGA-A6-3809-01 | 996 | 0 | 0.116484 |
| TCGA-AA-3492-01 | 1 | 1 | -0.05369 |
| TCGA-CM-5341-01 | 884 | 0 | -0.9951 |
| TCGA-CM-5862-01 | 153 | 0 | -0.82397 |
| TCGA-D5-6923-01 | 378 | 0 | -1.84968 |
| TCGA-A6-5667-01 | 887 | 0 | -1.24192 |
| TCGA-D5-6541-01 | 474 | 0 | -0.82244 |
| TCGA-AA-3811-01 | 306 | 0 | -0.55797 |
| TCGA-DM-A288-01 | 427 | 1 | -0.54009 |
| TCGA-CA-6717-01 | 388 | 0 | -1.35654 |
| TCGA-AA-3531-01 | 1035 | 0 | -0.42996 |
| TCGA-AA-3553-01 | 730 | 0 | -0.81933 |
| TCGA-A6-2681-01 | 1387 | 0 | -0.9596 |
| TCGA-AA-3976-01 | 791 | 0 | -0.37074 |
| TCGA-NH-A6GC-01 | 389 | 0 | -0.63169 |
| TCGA-AA-A02J-01 | 153 | 0 | -0.6344 |
| TCGA-AA-A00Q-01 | 1278 | 0 | -0.47495 |
| TCGA-A6-3807-01 | 1054 | 1 | -0.47043 |
| TCGA-AA-A004-01 | 424 | 0 | -0.36848 |
| TCGA-AA-3858-01 | 945 | 0 | -0.40451 |
| TCGA-AA-A02W-01 | 1247 | 0 | -0.40393 |
| TCGA-AA-3663-01 | 212 | 1 | -0.14598 |
| TCGA-CM-6163-01 | 427 | 0 | -1.43363 |
| TCGA-A6-5665-01 | 671 | 0 | -1.1796 |
| TCGA-CM-4751-01 | 822 | 0 | -0.38828 |
| TCGA-AA-3955-01 | 638 | 1 | -0.58922 |
| TCGA-AA-3819-01 | 761 | 0 | -0.55598 |
| TCGA-AA-3538-01 | 791 | 0 | -0.59768 |
| TCGA-AY-6196-01 | 6 | 1 | 4.140836 |
| TCGA-G4-6315-01 | 1883 | 0 | -0.99034 |
| TCGA-AD-A5EK-01 | 500 | 1 | -1.54542 |
| TCGA-WS-AB45-01 | 2130 | 0 | -2.89085 |
| TCGA-AZ-4315-01 | 1776 | 0 | -0.62073 |
| TCGA-DM-A280-01 | 236 | 0 | -0.06342 |
| TCGA-A6-4105-01 | 442 | 0 | -1.53144 |
| TCGA-AA-3856-01 | 30 | 0 | -0.48411 |
| TCGA-G4-6628-01 | 2424 | 1 | -0.89839 |
| TCGA-5M-AAT4-01 | 49 | 0 | -0.6508 |
| TCGA-RU-A8FL-01 | 1177 | 0 | -1.0417 |
| TCGA-AZ-4614-01 | 172 | 0 | -0.12568 |
| TCGA-DM-A28G-01 | 1849 | 1 | -0.88025 |
| TCGA-D5-6532-01 | 555 | 0 | -1.75426 |
| TCGA-AA-A01S-01 | 31 | 0 | -0.58025 |
| TCGA-CA-5254-01 | 386 | 0 | -1.41426 |
| TCGA-4T-AA8H-01 | 385 | 0 | -0.56921 |
| TCGA-AZ-6600-01 | 368 | 0 | -0.59564 |
| TCGA-AA-3952-01 | 61 | 0 | -0.91242 |
| TCGA-AA-A02F-01 | 1216 | 1 | -0.54298 |
| TCGA-AA-3860-01 | 945 | 0 | -0.97059 |
| TCGA-AA-A010-01 | 1064 | 1 | -0.14979 |
| TCGA-D5-6932-01 | 346 | 0 | -1.73698 |
| TCGA-AA-3519-01 | 276 | 1 | -0.57917 |
| TCGA-D5-5540-01 | 1706 | 0 | -1.69949 |
| TCGA-D5-6924-01 | 435 | 0 | -0.7434 |
| TCGA-AZ-4313-01 | 2310 | 0 | -0.29037 |
| TCGA-AA-3696-01 | 153 | 0 | -0.08786 |
| TCGA-DM-A28E-01 | 3648 | 1 | -1.07535 |
| TCGA-AA-3713-01 | 579 | 0 | -0.99241 |
| TCGA-A6-6141-01 | 255 | 0 | -0.73678 |
| TCGA-G4-6311-01 | 1199 | 0 | -1.56802 |
| TCGA-AD-6963-01 | 834 | 0 | -1.52959 |
| TCGA-AA-3855-01 | 975 | 1 | -0.42481 |
| TCGA-AA-A00N-01 | 122 | 1 | -0.51312 |
| TCGA-DM-A0XD-01 | 743 | 1 | -1.10614 |
| TCGA-CM-6679-01 | 306 | 0 | -1.74487 |
| TCGA-AA-3496-01 | 31 | 0 | -0.91699 |
| TCGA-AA-3562-01 | 608 | 1 | -0.52297 |
| TCGA-AZ-6603-01 | 899 | 1 | -1.3792 |
| TCGA-NH-A50V-01 | 588 | 0 | -1.40463 |
| TCGA-AA-3841-01 | 1124 | 0 | -0.45292 |
| TCGA-CA-6719-01 | 435 | 0 | -0.98399 |
| TCGA-F4-6856-01 | 1074 | 0 | -0.52802 |
| TCGA-CM-4746-01 | 1126 | 0 | -0.64868 |
| TCGA-CM-4748-01 | 792 | 0 | -0.04519 |
| TCGA-AA-3862-01 | 914 | 1 | -0.44457 |
| TCGA-AA-3989-01 | 242 | 0 | -0.57202 |
| TCGA-A6-2680-01 | 1068 | 0 | -0.90944 |
| TCGA-AY-5543-01 | 1004 | 0 | -1.86114 |
| TCGA-A6-2684-01 | 1127 | 1 | -0.50379 |
| TCGA-G4-6626-01 | 1 | 0 | -1.3862 |
| TCGA-AA-A01V-01 | 31 | 0 | -0.50313 |
| TCGA-AY-A71X-01 | 588 | 0 | -1.26877 |
| TCGA-DM-A1D6-01 | 570 | 0 | -2.20894 |
| TCGA-AD-5900-01 | 370 | 0 | -0.33182 |
| TCGA-G4-6303-01 | 2003 | 0 | -1.33989 |
| TCGA-D5-5539-01 | 596 | 0 | -1.28817 |
| TCGA-AA-3994-01 | 822 | 0 | -0.70471 |
| TCGA-AA-A03J-01 | 1246 | 1 | -0.48701 |
| TCGA-A6-2671-01 | 1331 | 0 | -0.77761 |
| TCGA-A6-6782-01 | 617 | 0 | -0.07654 |
| TCGA-AA-3968-01 | 669 | 1 | -0.55382 |
| TCGA-AA-3715-01 | 579 | 0 | -0.8529 |
| TCGA-SS-A7HO-01 | 1829 | 0 | -0.96343 |
| TCGA-G4-6588-01 | 796 | 0 | -0.9524 |
| TCGA-A6-A566-01 | 758 | 0 | -0.9698 |
| TCGA-NH-A50U-01 | 334 | 0 | 0.374203 |
| TCGA-AY-A8YK-01 | 573 | 1 | -1.27382 |
| TCGA-A6-6140-01 | 734 | 0 | -1.59522 |
| TCGA-QL-A97D-01 | 666 | 1 | -1.10333 |
| TCGA-CK-4951-01 | 1492 | 0 | -0.77269 |
| TCGA-AA-3854-01 | 1096 | 1 | -0.70975 |
| TCGA-AA-3560-01 | 608 | 1 | -0.41765 |
| TCGA-G4-6310-01 | 1935 | 0 | -0.87132 |
| TCGA-A6-A567-01 | 1881 | 0 | -0.48292 |
| TCGA-CM-6171-01 | 427 | 0 | -1.37623 |
| TCGA-CM-5864-01 | 457 | 0 | -0.74873 |
| TCGA-AA-3846-01 | 518 | 0 | -0.95865 |
| TCGA-AA-3692-01 | 1095 | 0 | -1.07352 |
| TCGA-AA-3678-01 | 1430 | 0 | -0.9201 |
| TCGA-AY-6197-01 | 652 | 0 | -1.44663 |
| TCGA-A6-5662-01 | 718 | 0 | -1.47682 |
| TCGA-AA-3530-01 | 580 | 0 | -0.50474 |
| TCGA-F4-6463-01 | 1087 | 0 | -0.98739 |
| TCGA-CK-5912-01 | 1493 | 0 | -1.34619 |
| TCGA-AA-A02K-01 | 426 | 0 | -1.01586 |
| TCGA-DM-A28C-01 | 2475 | 0 | -1.28085 |
| TCGA-CM-5348-01 | 699 | 1 | -0.41698 |
| TCGA-AM-5821-01 | 28 | 0 | -2.87629 |
| TCGA-DM-A28H-01 | 3561 | 0 | -0.95063 |
| TCGA-AD-6889-01 | 2532 | 0 | -1.42007 |
| TCGA-AZ-6608-01 | 59 | 1 | -1.40214 |
| TCGA-F4-6570-01 | 188 | 1 | 0.403686 |
| TCGA-AD-6964-01 | 331 | 1 | 0.269141 |
| TCGA-AA-3947-01 | 1004 | 1 | -0.50854 |
| TCGA-F4-6805-01 | 1047 | 0 | -0.94049 |
| TCGA-D5-6529-01 | 614 | 0 | -1.32225 |
| TCGA-AY-6386-01 | 542 | 0 | -0.79138 |
| TCGA-AA-3667-01 | 426 | 0 | -0.49303 |
| TCGA-5M-AAT6-01 | 290 | 0 | -0.40754 |
| TCGA-AA-3833-01 | 485 | 0 | -0.42328 |
| TCGA-AA-3518-01 | 31 | 0 | -0.12491 |
| TCGA-AA-3877-01 | 943 | 1 | -0.68086 |
| TCGA-A6-6652-01 | 751 | 1 | -0.99604 |
| TCGA-D5-6536-01 | 543 | 0 | -1.42381 |
| TCGA-AA-3869-01 | 822 | 0 | -0.61226 |
| TCGA-AA-3710-01 | 821 | 0 | -0.17954 |
| TCGA-AA-3977-01 | 761 | 0 | -0.38945 |
| TCGA-AA-A00W-01 | 456 | 0 | -0.73437 |
| TCGA-AA-3522-01 | 1127 | 0 | -0.50496 |
| TCGA-AA-3861-01 | 914 | 0 | -0.47789 |
| TCGA-A6-5664-01 | 672 | 0 | -1.51426 |
| TCGA-AA-3526-01 | 580 | 1 | -0.69356 |
| TCGA-A6-2685-01 | 1133 | 1 | 0.182425 |
| TCGA-AA-3867-01 | 731 | 0 | -0.33987 |
| TCGA-AA-3488-01 | 153 | 0 | -0.84722 |
| TCGA-CA-6718-01 | 306 | 1 | 1.413851 |
| TCGA-G4-6320-01 | 804 | 1 | -0.53041 |
| TCGA-D5-6538-01 | 521 | 1 | -0.64113 |
| TCGA-AA-3524-01 | 1096 | 1 | -0.21325 |
| TCGA-A6-3808-01 | 1014 | 0 | -0.66507 |
| TCGA-QG-A5YW-01 | 896 | 0 | -1.81154 |
| TCGA-AY-A54L-01 | 525 | 0 | -1.17067 |
| TCGA-CA-5255-01 | 376 | 1 | -0.66094 |
| TCGA-AZ-5403-01 | 1910 | 0 | -1.86716 |
| TCGA-AZ-6601-01 | 3042 | 1 | -0.40544 |
| TCGA-CK-4947-01 | 534 | 0 | -1.24533 |
| TCGA-AA-A02Y-01 | 1216 | 0 | -0.87118 |
| TCGA-AA-3975-01 | 1036 | 0 | -0.46887 |
| TCGA-CK-6748-01 | 61 | 0 | -2.08771 |
| TCGA-G4-6306-01 | 1359 | 0 | -0.46651 |
| TCGA-QG-A5Z2-01 | 952 | 0 | -1.66563 |
| TCGA-AA-3494-01 | 31 | 1 | -0.49232 |
| TCGA-AA-3543-01 | 30 | 1 | 0.2941 |
| TCGA-AA-A01C-01 | 457 | 0 | -0.32988 |
| TCGA-CK-4952-01 | 475 | 1 | -1.07577 |
| TCGA-D5-6531-01 | 540 | 0 | -0.46266 |
| TCGA-AA-A00A-01 | 1157 | 0 | -0.22675 |
| TCGA-D5-5538-01 | 1661 | 0 | -0.75857 |
| TCGA-A6-5660-01 | 888 | 1 | -1.28751 |
| TCGA-AA-3842-01 | 1126 | 0 | -0.15488 |
| TCGA-AZ-6605-01 | 159 | 0 | -1.58447 |
| TCGA-CM-5349-01 | 915 | 0 | -1.06378 |
| TCGA-AU-3779-01 | 441 | 0 | -1.62036 |
| TCGA-A6-6137-01 | 824 | 0 | -1.15545 |
| TCGA-F4-6854-01 | 16 | 0 | -0.96017 |
| TCGA-AA-3675-01 | 1431 | 0 | -1.59179 |
| TCGA-NH-A6GA-01 | 302 | 0 | -0.95988 |
| TCGA-AA-A01Z-01 | 1126 | 0 | -0.36098 |
| TCGA-CM-6172-01 | 335 | 0 | -1.49278 |
| TCGA-5M-AATE-01 | 1200 | 0 | -0.99829 |
| TCGA-D5-6928-01 | 354 | 0 | -0.69469 |
| TCGA-DM-A1D9-01 | 4270 | 0 | -2.06162 |
| TCGA-AA-A01I-01 | 943 | 0 | -0.71009 |
| TCGA-D5-6930-01 | 406 | 0 | -0.92878 |
| TCGA-AA-3555-01 | 911 | 0 | -0.71269 |
| TCGA-CM-6676-01 | 337 | 0 | -1.03336 |
| TCGA-A6-4107-01 | 987 | 0 | -0.95956 |
| TCGA-AA-A01R-01 | 1065 | 1 | 0.28205 |
| TCGA-G4-6294-01 | 858 | 0 | -0.97499 |
| TCGA-AZ-4684-01 | 1977 | 0 | -0.45506 |
| TCGA-AA-A01X-01 | 791 | 0 | -0.61999 |
| TCGA-CM-4750-01 | 244 | 0 | -0.6332 |
| TCGA-AA-A01K-01 | 943 | 1 | -0.16414 |
| TCGA-G4-6314-01 | 1093 | 0 | -1.43803 |
| TCGA-CA-5796-01 | 377 | 1 | -0.60067 |
| TCGA-AZ-6599-01 | 206 | 0 | -1.15297 |
| TCGA-AD-6901-01 | 682 | 0 | 0.102566 |
| TCGA-G4-6299-01 | 2268 | 1 | -0.19918 |
| TCGA-F4-6808-01 | 1024 | 0 | -1.26072 |
| TCGA-AA-3516-01 | 396 | 1 | -0.36355 |
| TCGA-A6-5666-01 | 995 | 0 | -0.90208 |
| TCGA-AA-3510-01 | 1946 | 0 | -0.56469 |
| TCGA-G4-6293-01 | 4051 | 0 | -1.10109 |
| TCGA-AD-6895-01 | 763 | 0 | -1.12734 |
| TCGA-AA-A02E-01 | 90 | 0 | -0.3428 |
| TCGA-DM-A28A-01 | 805 | 0 | -0.91849 |
| TCGA-A6-6654-01 | 726 | 0 | -1.84096 |
| TCGA-CA-5797-01 | 383 | 1 | -0.499 |
| TCGA-CK-6747-01 | 820 | 0 | -1.20108 |
| TCGA-AA-A01Q-01 | 31 | 1 | -0.19729 |
| TCGA-AA-3848-01 | 306 | 0 | -0.39511 |
| TCGA-AZ-6606-01 | 357 | 0 | -1.31801 |
| TCGA-AZ-4308-01 | 3324 | 0 | -0.80759 |
| TCGA-AA-A00K-01 | 549 | 1 | -0.45699 |
| TCGA-AA-3534-01 | 882 | 0 | -0.31054 |
| TCGA-AA-A02R-01 | 670 | 1 | 0.403512 |
| TCGA-CM-5861-01 | 457 | 0 | -0.5942 |
| TCGA-AA-3502-01 | 1065 | 0 | -0.30198 |
| TCGA-AA-3697-01 | 2587 | 0 | -1.10848 |
| TCGA-DM-A1HA-01 | 2600 | 0 | -1.81045 |
| TCGA-AA-A017-01 | 457 | 0 | -0.44853 |
| TCGA-CM-6170-01 | 457 | 0 | -1.53014 |
| TCGA-AA-3542-01 | 395 | 0 | 0.220213 |
| TCGA-AA-3812-01 | 1066 | 0 | -0.51442 |
| TCGA-AZ-6607-01 | 97 | 0 | -1.79679 |
| TCGA-G4-6323-01 | 419 | 0 | -0.89316 |
| TCGA-AA-3971-01 | 489 | 0 | -0.50585 |
| TCGA-F4-6460-01 | 972 | 1 | -1.00081 |
| TCGA-A6-A56B-01 | 1711 | 0 | -0.44608 |
| TCGA-AA-A00E-01 | 913 | 0 | 0.005506 |

**Supplementary Table 2. Riskscore of 427 enrolled patients’ overall survival model.**

| Tag | Time | Status | RiskScore |
| --- | --- | --- | --- |
| TCGA-AA-3979-01 | 730 | 0 | 0.992542 |
| TCGA-AA-3655-01 | 1856 | 0 | 0.443371 |
| TCGA-D5-6535-01 | 460 | 0 | 0.381884 |
| TCGA-CK-4950-01 | 2599 | 0 | 0.513013 |
| TCGA-A6-3810-01 | 1111 | 0 | 0.028354 |
| TCGA-AD-6888-01 | 343 | 1 | 0.233934 |
| TCGA-AA-3680-01 | 335 | 0 | 0.124137 |
| TCGA-3L-AA1B-01 | 475 | 0 | 0.342492 |
| TCGA-AY-4070-01 | 186 | 1 | 0.363543 |
| TCGA-CM-6675-01 | 337 | 1 | 0.57766 |
| TCGA-A6-6650-01 | 627 | 0 | -0.38681 |
| TCGA-D5-6898-01 | 229 | 0 | 0.46508 |
| TCGA-QG-A5Z1-01 | 256 | 0 | 0.478631 |
| TCGA-AA-A00J-01 | 549 | 0 | 0.408468 |
| TCGA-A6-6138-01 | 685 | 0 | 0.197207 |
| TCGA-NH-A8F8-01 | 511 | 0 | 0.641848 |
| TCGA-CK-6751-01 | 518 | 0 | 0.219799 |
| TCGA-D5-6534-01 | 1316 | 0 | 0.297836 |
| TCGA-A6-2677-01 | 740 | 0 | 0.162863 |
| TCGA-T9-A92H-01 | 81 | 1 | 0.286881 |
| TCGA-AA-3506-01 | 1765 | 0 | 0.066369 |
| TCGA-AD-6890-01 | 746 | 0 | 0.240607 |
| TCGA-DM-A1D4-01 | 1920 | 1 | 0.111071 |
| TCGA-AA-A00F-01 | 1035 | 0 | 0.163868 |
| TCGA-A6-6780-01 | 612 | 0 | 0.128266 |
| TCGA-AA-3939-01 | 395 | 0 | 0.106037 |
| TCGA-AA-3679-01 | 457 | 0 | 0.02951 |
| TCGA-DM-A285-01 | 179 | 0 | 0.580069 |
| TCGA-NH-A6GB-01 | 476 | 0 | 0.241189 |
| TCGA-AA-3972-01 | 1216 | 1 | 0.116991 |
| TCGA-A6-2675-01 | 1321 | 0 | -0.42617 |
| TCGA-G4-6321-01 | 672 | 0 | -0.13035 |
| TCGA-CK-5916-01 | 292 | 1 | 0.28446 |
| TCGA-DM-A28M-01 | 2895 | 0 | -0.00762 |
| TCGA-CM-5344-01 | 670 | 0 | 0.341803 |
| TCGA-AA-3561-01 | 424 | 0 | 0.013593 |
| TCGA-AD-6965-01 | 648 | 1 | 0.370522 |
| TCGA-QG-A5YV-01 | 1301 | 0 | 0.116861 |
| TCGA-A6-5656-01 | 1001 | 0 | 0.43324 |
| TCGA-AD-6548-01 | 650 | 0 | 0.172723 |
| TCGA-A6-6651-01 | 662 | 0 | 0.429471 |
| TCGA-CM-6166-01 | 669 | 0 | 0.546834 |
| TCGA-CM-6162-01 | 365 | 0 | 0.507793 |
| TCGA-AA-3870-01 | 912 | 0 | 0.308373 |
| TCGA-AA-A00R-01 | 30 | 0 | -0.04131 |
| TCGA-D5-5537-01 | 722 | 1 | 0.228227 |
| TCGA-AA-3509-01 | 1915 | 0 | -0.05189 |
| TCGA-AA-3815-01 | 1005 | 0 | -0.14309 |
| TCGA-F4-6806-01 | 336 | 1 | 0.127798 |
| TCGA-A6-6142-01 | 654 | 1 | 0.869368 |
| TCGA-AA-3544-01 | 426 | 0 | 0.020016 |
| TCGA-DM-A1DB-01 | 1348 | 0 | 0.658832 |
| TCGA-A6-2678-01 | 1286 | 0 | 0.172845 |
| TCGA-AA-3664-01 | 1643 | 0 | -0.09172 |
| TCGA-AA-3660-01 | 2375 | 0 | 0.031821 |
| TCGA-AA-3666-01 | 61 | 0 | -0.11778 |
| TCGA-CM-6678-01 | 1 | 1 | 0.580507 |
| TCGA-A6-2672-01 | 1419 | 0 | 0.178165 |
| TCGA-CM-5868-01 | 518 | 0 | 0.440815 |
| TCGA-A6-2686-01 | 1126 | 0 | -0.50014 |
| TCGA-A6-2682-01 | 381 | 1 | 0.06607 |
| TCGA-G4-6304-01 | 859 | 1 | 0.133411 |
| TCGA-CK-5914-01 | 669 | 0 | -0.15927 |
| TCGA-D5-5541-01 | 1701 | 0 | -0.14074 |
| TCGA-AA-A00U-01 | 518 | 0 | 0.009187 |
| TCGA-AA-3532-01 | 882 | 0 | 0.018374 |
| TCGA-DM-A1D8-01 | 383 | 0 | 0.22241 |
| TCGA-AM-5820-01 | 14 | 0 | 0.370341 |
| TCGA-F4-6855-01 | 1442 | 0 | 0.231393 |
| TCGA-A6-6781-01 | 598 | 0 | -0.3849 |
| TCGA-CA-6716-01 | 371 | 0 | 0.602158 |
| TCGA-D5-6927-01 | 287 | 0 | 0.128933 |
| TCGA-A6-6653-01 | 742 | 0 | 0.164332 |
| TCGA-AA-3864-01 | 1612 | 0 | 0.127763 |
| TCGA-DM-A1DA-01 | 228 | 0 | 0.50454 |
| TCGA-F4-6704-01 | 47 | 0 | -0.51966 |
| TCGA-AA-3552-01 | 122 | 1 | 0.105187 |
| TCGA-AA-A02H-01 | 61 | 0 | -0.04587 |
| TCGA-DM-A28K-01 | 2988 | 0 | 0.326679 |
| TCGA-AZ-4323-01 | 43 | 0 | 0.140804 |
| TCGA-A6-A565-01 | 301 | 1 | 0.065104 |
| TCGA-F4-6459-01 | 262 | 0 | 0.741322 |
| TCGA-G4-6317-01 | 592 | 1 | -0.05493 |
| TCGA-AA-3520-01 | 731 | 0 | 0.175372 |
| TCGA-AA-3941-01 | 730 | 0 | 0.036227 |
| TCGA-F4-6569-01 | 1087 | 0 | 0.380833 |
| TCGA-D5-6926-01 | 275 | 0 | 0.221342 |
| TCGA-G4-6297-01 | 439 | 1 | 1.183382 |
| TCGA-QG-A5YX-01 | 1003 | 0 | -0.20403 |
| TCGA-A6-A5ZU-01 | 293 | 0 | 0.159621 |
| TCGA-AY-A69D-01 | 543 | 0 | 0.583926 |
| TCGA-A6-6649-01 | 735 | 0 | 0.433535 |
| TCGA-CK-5913-01 | 1561 | 0 | 0.182813 |
| TCGA-4N-A93T-01 | 146 | 0 | 0.477336 |
| TCGA-AA-3821-01 | 31 | 0 | 0.214312 |
| TCGA-AA-3970-01 | 1096 | 0 | 0.472279 |
| TCGA-AA-3525-01 | 1 | 0 | 0.060206 |
| TCGA-CM-6167-01 | 456 | 0 | 0.323195 |
| TCGA-A6-5661-01 | 1020 | 0 | 0.03288 |
| TCGA-AA-A01P-01 | 762 | 1 | 0.564316 |
| TCGA-D5-6539-01 | 380 | 0 | -0.14417 |
| TCGA-AA-3818-01 | 30 | 0 | 0.188935 |
| TCGA-AA-3681-01 | 182 | 0 | 0.04276 |
| TCGA-G4-6625-01 | 2564 | 1 | 0.183283 |
| TCGA-AA-3514-01 | 31 | 0 | 0.183853 |
| TCGA-F4-6809-01 | 403 | 0 | 0.268709 |
| TCGA-AA-3966-01 | 61 | 0 | 0.904778 |
| TCGA-AA-A00L-01 | 1157 | 0 | 0.120367 |
| TCGA-AA-3950-01 | 730 | 0 | 0.060536 |
| TCGA-AA-3866-01 | 518 | 0 | -0.13858 |
| TCGA-CM-6674-01 | 394 | 0 | 0.455662 |
| TCGA-A6-2676-01 | 1305 | 0 | 0.008516 |
| TCGA-AA-3548-01 | 1034 | 0 | 0.206106 |
| TCGA-CK-4948-01 | 4502 | 0 | 0.735061 |
| TCGA-G4-6627-01 | 1003 | 1 | 0.659968 |
| TCGA-AA-3844-01 | 365 | 1 | -0.02132 |
| TCGA-A6-5657-01 | 962 | 0 | 0.558996 |
| TCGA-AU-6004-01 | 824 | 0 | -0.17333 |
| TCGA-D5-6922-01 | 308 | 0 | 0.151187 |
| TCGA-AA-3662-01 | 184 | 0 | 0.267484 |
| TCGA-G4-6307-01 | 1674 | 0 | 0.03637 |
| TCGA-A6-6648-01 | 686 | 1 | 0.650352 |
| TCGA-A6-2674-01 | 735 | 1 | -0.05811 |
| TCGA-CM-6168-01 | 395 | 0 | 0.427126 |
| TCGA-CM-4752-01 | 396 | 0 | -0.0909 |
| TCGA-DM-A1D0-01 | 3974 | 0 | 0.256357 |
| TCGA-AA-A00Z-01 | 669 | 0 | 0.097712 |
| TCGA-CM-4743-01 | 701 | 0 | 0.072525 |
| TCGA-AA-3930-01 | 61 | 0 | 0.157868 |
| TCGA-AA-A02O-01 | 28 | 0 | 0.221022 |
| TCGA-AA-3831-01 | 547 | 0 | -0.00431 |
| TCGA-A6-5659-01 | 926 | 0 | 0.110521 |
| TCGA-AA-3973-01 | 397 | 0 | 0.349327 |
| TCGA-CM-4744-01 | 609 | 0 | 0.237938 |
| TCGA-D5-6929-01 | 408 | 0 | -0.08633 |
| TCGA-AA-3982-01 | 822 | 0 | 0.118856 |
| TCGA-AA-3554-01 | 546 | 0 | 0.084658 |
| TCGA-AZ-4616-01 | 156 | 0 | 0.768396 |
| TCGA-AA-3956-01 | 1035 | 0 | 0.093729 |
| TCGA-D5-6931-01 | 365 | 0 | 0.246698 |
| TCGA-AA-A01D-01 | 183 | 1 | 0.119984 |
| TCGA-AA-3986-01 | 580 | 0 | -0.16321 |
| TCGA-DM-A28F-01 | 1094 | 0 | 0.60962 |
| TCGA-DM-A0X9-01 | 3641 | 0 | 0.105953 |
| TCGA-AA-3837-01 | 1186 | 0 | 0.068901 |
| TCGA-AA-3495-01 | 1127 | 0 | -0.1263 |
| TCGA-F4-6703-01 | 1456 | 0 | 0.558439 |
| TCGA-AA-A00D-01 | 578 | 0 | -0.08267 |
| TCGA-AA-3685-01 | 1127 | 0 | -0.40862 |
| TCGA-AZ-4615-01 | 688 | 1 | 1.206821 |
| TCGA-NH-A8F7-01 | 543 | 0 | -0.11694 |
| TCGA-AA-A024-01 | 1188 | 0 | 0.195959 |
| TCGA-NH-A50T-01 | 553 | 0 | 0.121563 |
| TCGA-CM-5860-01 | 974 | 0 | -0.09006 |
| TCGA-CA-5256-01 | 379 | 0 | 0.015519 |
| TCGA-CM-6680-01 | 366 | 0 | 0.05304 |
| TCGA-CA-6715-01 | 383 | 0 | 0.362438 |
| TCGA-G4-6322-01 | 789 | 1 | 0.265323 |
| TCGA-AA-3511-01 | 59 | 1 | 0.880431 |
| TCGA-D5-6540-01 | 491 | 0 | 0.179501 |
| TCGA-D5-6920-01 | 377 | 0 | -0.0758 |
| TCGA-G4-6586-01 | 1089 | 0 | 0.271111 |
| TCGA-AA-A029-01 | 1581 | 0 | 0.062718 |
| TCGA-AA-3875-01 | 549 | 0 | 0.023809 |
| TCGA-CM-6677-01 | 337 | 0 | 0.197477 |
| TCGA-AA-3688-01 | 578 | 0 | 0.232757 |
| TCGA-AA-A01T-01 | 1005 | 0 | 0.175192 |
| TCGA-G4-6295-01 | 254 | 0 | 0.24929 |
| TCGA-F4-6807-01 | 1309 | 0 | 0.442628 |
| TCGA-AZ-6598-01 | 1503 | 0 | -0.13428 |
| TCGA-AA-A01G-01 | 365 | 0 | 0.001484 |
| TCGA-AA-A01F-01 | 974 | 0 | 0.091281 |
| TCGA-CM-6161-01 | 457 | 0 | -0.63257 |
| TCGA-AA-3549-01 | 639 | 0 | 0.522605 |
| TCGA-CM-6164-01 | 883 | 0 | 0.270164 |
| TCGA-D5-6537-01 | 123 | 1 | 0.263495 |
| TCGA-D5-6533-01 | 775 | 0 | 0.326653 |
| TCGA-A6-2683-01 | 300 | 1 | 0.146169 |
| TCGA-CM-4747-01 | 1 | 1 | 0.355175 |
| TCGA-AA-3949-01 | 791 | 0 | 0.087626 |
| TCGA-AZ-4682-01 | 680 | 0 | 0.038726 |
| TCGA-AD-6899-01 | 176 | 0 | -0.11971 |
| TCGA-CM-6169-01 | 396 | 0 | -0.0244 |
| TCGA-AA-3673-01 | 1522 | 0 | 0.113288 |
| TCGA-CM-5863-01 | 457 | 0 | 0.642241 |
| TCGA-AA-3980-01 | 242 | 0 | -0.25726 |
| TCGA-AZ-5407-01 | 2683 | 0 | 0.077523 |
| TCGA-D5-7000-01 | 312 | 0 | 0.230575 |
| TCGA-F4-6461-01 | 288 | 1 | 1.176178 |
| TCGA-CM-6165-01 | 488 | 0 | -0.15894 |
| TCGA-AA-3489-01 | 214 | 0 | 0.211531 |
| TCGA-AA-3517-01 | 821 | 1 | 0.155608 |
| TCGA-DM-A282-01 | 4233 | 0 | 0.539153 |
| TCGA-D5-6530-01 | 621 | 0 | -0.14056 |
| TCGA-AA-A00O-01 | 822 | 0 | 0.482049 |
| TCGA-A6-2679-01 | 1366 | 0 | 0.078124 |
| TCGA-AA-3851-01 | 1006 | 0 | 0.068799 |
| TCGA-A6-3809-01 | 996 | 0 | -0.11193 |
| TCGA-AA-3492-01 | 1 | 0 | 0.0157 |
| TCGA-CM-5341-01 | 884 | 0 | 0.074786 |
| TCGA-CM-5862-01 | 31 | 1 | 0.510484 |
| TCGA-D5-6923-01 | 378 | 0 | 0.610456 |
| TCGA-A6-5667-01 | 887 | 0 | 0.453304 |
| TCGA-D5-6541-01 | 474 | 0 | 0.115084 |
| TCGA-AA-3811-01 | 306 | 0 | 0.169646 |
| TCGA-DM-A288-01 | 320 | 1 | 0.282074 |
| TCGA-CA-6717-01 | 388 | 0 | 0.287726 |
| TCGA-AA-3531-01 | 1035 | 0 | 0.024276 |
| TCGA-AA-3553-01 | 730 | 0 | 0.07032 |
| TCGA-A6-2681-01 | 1150 | 1 | 0.263941 |
| TCGA-AA-3976-01 | 791 | 0 | 0.097822 |
| TCGA-NH-A6GC-01 | 389 | 0 | 0.191856 |
| TCGA-AA-A02J-01 | 153 | 0 | 0.014756 |
| TCGA-AA-A00Q-01 | 1278 | 0 | 0.22201 |
| TCGA-A6-3807-01 | 1054 | 0 | 0.118567 |
| TCGA-AA-A004-01 | 424 | 0 | 0.092628 |
| TCGA-AA-3858-01 | 945 | 0 | 0.03554 |
| TCGA-AA-A02W-01 | 1005 | 1 | 0.421709 |
| TCGA-AA-3663-01 | 212 | 0 | 0.124241 |
| TCGA-CM-6163-01 | 427 | 0 | 0.210661 |
| TCGA-A6-5665-01 | 529 | 1 | 0.048377 |
| TCGA-CM-4751-01 | 822 | 0 | 0.106524 |
| TCGA-AA-3955-01 | 638 | 0 | 0.130053 |
| TCGA-AA-3819-01 | 761 | 0 | 0.014811 |
| TCGA-AA-3538-01 | 791 | 0 | -0.39735 |
| TCGA-AY-6196-01 | 6 | 0 | 0.235455 |
| TCGA-G4-6315-01 | 1883 | 0 | 0.092076 |
| TCGA-AD-A5EK-01 | 500 | 0 | 0.453397 |
| TCGA-WS-AB45-01 | 2130 | 0 | 0.671078 |
| TCGA-AZ-4315-01 | 1776 | 0 | 0.026532 |
| TCGA-DM-A280-01 | 236 | 0 | 0.151589 |
| TCGA-A6-4105-01 | 364 | 1 | 0.341262 |
| TCGA-AA-3856-01 | 30 | 0 | 0.229201 |
| TCGA-G4-6628-01 | 2424 | 0 | 0.369059 |
| TCGA-5M-AAT4-01 | 49 | 0 | 0.250365 |
| TCGA-RU-A8FL-01 | 256 | 1 | 0.585669 |
| TCGA-AZ-4614-01 | 172 | 0 | 0.077997 |
| TCGA-DM-A28G-01 | 1849 | 0 | -0.05095 |
| TCGA-D5-6532-01 | 555 | 0 | 0.572387 |
| TCGA-AA-A01S-01 | 31 | 0 | 0.091039 |
| TCGA-CA-5254-01 | 386 | 0 | -0.3587 |
| TCGA-4T-AA8H-01 | 385 | 0 | 0.177 |
| TCGA-AZ-6600-01 | 368 | 0 | 0.492419 |
| TCGA-AA-3952-01 | 61 | 0 | -0.03968 |
| TCGA-AA-A02F-01 | 1216 | 0 | 0.19978 |
| TCGA-AA-3860-01 | 945 | 0 | 0.127181 |
| TCGA-AA-A010-01 | 1064 | 0 | 0.043291 |
| TCGA-D5-6932-01 | 346 | 0 | 0.461385 |
| TCGA-AA-3519-01 | 276 | 0 | 0.061693 |
| TCGA-D5-5540-01 | 1706 | 0 | 0.575414 |
| TCGA-D5-6924-01 | 435 | 0 | 0.148237 |
| TCGA-AZ-4313-01 | 2310 | 0 | 0.0353 |
| TCGA-AA-3696-01 | 153 | 0 | 0.0221 |
| TCGA-DM-A28E-01 | 3648 | 0 | 0.29267 |
| TCGA-AA-3713-01 | 579 | 0 | 0.629624 |
| TCGA-A6-6141-01 | 255 | 0 | 0.209251 |
| TCGA-G4-6311-01 | 1199 | 0 | 0.357053 |
| TCGA-AD-6963-01 | 834 | 0 | -0.2007 |
| TCGA-AA-3855-01 | 975 | 0 | -0.05117 |
| TCGA-AA-A00N-01 | 122 | 0 | 0.211398 |
| TCGA-DM-A0XD-01 | 484 | 1 | 0.381078 |
| TCGA-CM-6679-01 | 306 | 0 | 0.118501 |
| TCGA-AA-3496-01 | 31 | 0 | -0.73218 |
| TCGA-AA-3562-01 | 608 | 0 | 0.074291 |
| TCGA-AZ-6603-01 | 899 | 0 | 0.120538 |
| TCGA-NH-A50V-01 | 588 | 0 | 0.373706 |
| TCGA-AA-3841-01 | 1124 | 0 | 0.085064 |
| TCGA-CA-6719-01 | 354 | 1 | 0.829837 |
| TCGA-F4-6856-01 | 1074 | 0 | 0.133124 |
| TCGA-CM-4746-01 | 1126 | 0 | 0.197035 |
| TCGA-CM-4748-01 | 792 | 0 | 0.006564 |
| TCGA-AA-3862-01 | 914 | 0 | -0.11512 |
| TCGA-AA-3989-01 | 242 | 0 | 0.14091 |
| TCGA-A6-2680-01 | 1068 | 0 | 0.117066 |
| TCGA-AY-5543-01 | 1004 | 0 | -0.22237 |
| TCGA-A6-2684-01 | 976 | 1 | 0.144923 |
| TCGA-G4-6626-01 | 1 | 0 | 0.179493 |
| TCGA-AA-A01V-01 | 31 | 0 | 0.051088 |
| TCGA-AY-A71X-01 | 588 | 0 | 0.340051 |
| TCGA-DM-A1D6-01 | 570 | 0 | 0.349677 |
| TCGA-AD-5900-01 | 370 | 0 | 0.598542 |
| TCGA-G4-6303-01 | 872 | 1 | 1.179351 |
| TCGA-D5-5539-01 | 579 | 1 | 0.597038 |
| TCGA-AA-3994-01 | 822 | 0 | 0.302811 |
| TCGA-AA-A03J-01 | 1246 | 0 | 0.238498 |
| TCGA-A6-2671-01 | 535 | 1 | 0.304151 |
| TCGA-A6-6782-01 | 617 | 0 | 0.349591 |
| TCGA-AA-3968-01 | 669 | 0 | 0.083856 |
| TCGA-AA-3715-01 | 153 | 1 | 0.21007 |
| TCGA-SS-A7HO-01 | 506 | 1 | 0.444282 |
| TCGA-G4-6588-01 | 796 | 0 | 0.253879 |
| TCGA-A6-A566-01 | 257 | 1 | 0.297201 |
| TCGA-NH-A50U-01 | 276 | 1 | 1.435397 |
| TCGA-AY-A8YK-01 | 573 | 0 | 0.316593 |
| TCGA-A6-6140-01 | 734 | 0 | -0.26678 |
| TCGA-QL-A97D-01 | 666 | 0 | 0.335108 |
| TCGA-CK-4951-01 | 480 | 1 | 0.387363 |
| TCGA-AA-3854-01 | 1096 | 0 | 0.167734 |
| TCGA-AA-3560-01 | 608 | 0 | -0.00261 |
| TCGA-G4-6310-01 | 1935 | 0 | 0.291523 |
| TCGA-A6-A567-01 | 612 | 1 | 0.216872 |
| TCGA-CM-6171-01 | 427 | 0 | 0.829379 |
| TCGA-CM-5864-01 | 457 | 0 | -0.06946 |
| TCGA-AA-3846-01 | 518 | 0 | 0.262512 |
| TCGA-AA-3692-01 | 396 | 1 | -0.14887 |
| TCGA-AA-3678-01 | 1430 | 0 | -0.22435 |
| TCGA-AY-6197-01 | 652 | 0 | 0.307701 |
| TCGA-A6-5662-01 | 495 | 1 | 1.158013 |
| TCGA-AA-3530-01 | 580 | 0 | 0.052985 |
| TCGA-F4-6463-01 | 1087 | 0 | -0.42773 |
| TCGA-CK-5912-01 | 1493 | 0 | -0.39785 |
| TCGA-AA-A02K-01 | 306 | 1 | 0.713942 |
| TCGA-DM-A28C-01 | 1929 | 1 | 0.624299 |
| TCGA-CM-5348-01 | 699 | 0 | 0.111214 |
| TCGA-AM-5821-01 | 28 | 0 | 0.003642 |
| TCGA-DM-A28H-01 | 484 | 1 | 0.862336 |
| TCGA-AD-6889-01 | 1516 | 1 | 0.494994 |
| TCGA-AZ-6608-01 | 59 | 0 | 0.213036 |
| TCGA-F4-6570-01 | 188 | 0 | 0.461781 |
| TCGA-AD-6964-01 | 325 | 1 | 0.030147 |
| TCGA-AA-3947-01 | 1004 | 0 | 0.011267 |
| TCGA-F4-6805-01 | 1047 | 0 | 0.150894 |
| TCGA-D5-6529-01 | 386 | 1 | 0.318609 |
| TCGA-AY-6386-01 | 542 | 0 | 0.166416 |
| TCGA-AA-3667-01 | 426 | 0 | 0.04471 |
| TCGA-5M-AAT6-01 | 219 | 1 | -0.07836 |
| TCGA-AA-3833-01 | 485 | 0 | 0.102231 |
| TCGA-AA-3518-01 | 31 | 0 | 0.132734 |
| TCGA-AA-3877-01 | 943 | 0 | 0.073481 |
| TCGA-A6-6652-01 | 751 | 0 | 0.580822 |
| TCGA-D5-6536-01 | 493 | 1 | 0.916845 |
| TCGA-AA-3869-01 | 822 | 0 | 0.09282 |
| TCGA-AA-3710-01 | 821 | 0 | 0.045676 |
| TCGA-AA-3977-01 | 761 | 0 | -0.02499 |
| TCGA-AA-A00W-01 | 456 | 0 | 0.165278 |
| TCGA-AA-3522-01 | 1127 | 0 | -0.00493 |
| TCGA-AA-3861-01 | 914 | 0 | 0.057098 |
| TCGA-A6-5664-01 | 183 | 1 | 0.485347 |
| TCGA-AA-3526-01 | 580 | 0 | -0.25963 |
| TCGA-A6-2685-01 | 948 | 1 | 0.458138 |
| TCGA-AA-3867-01 | 394 | 1 | 0.211025 |
| TCGA-AA-3488-01 | 153 | 0 | 0.082725 |
| TCGA-CA-6718-01 | 207 | 1 | 0.788969 |
| TCGA-G4-6320-01 | 804 | 0 | 0.022272 |
| TCGA-D5-6538-01 | 521 | 0 | -0.10221 |
| TCGA-AA-3524-01 | 1096 | 0 | -0.01579 |
| TCGA-A6-3808-01 | 1014 | 0 | 0.120781 |
| TCGA-QG-A5YW-01 | 896 | 0 | 0.808448 |
| TCGA-AY-A54L-01 | 396 | 1 | 0.945007 |
| TCGA-CA-5255-01 | 376 | 0 | 0.331235 |
| TCGA-AZ-5403-01 | 304 | 1 | 0.493856 |
| TCGA-AZ-6601-01 | 2270 | 1 | -0.01143 |
| TCGA-CK-4947-01 | 21 | 1 | 0.303115 |
| TCGA-AA-A02Y-01 | 1216 | 0 | 0.426723 |
| TCGA-AA-3975-01 | 1036 | 0 | 0.188396 |
| TCGA-CK-6748-01 | 61 | 0 | 1.157296 |
| TCGA-G4-6306-01 | 1359 | 0 | 0.090628 |
| TCGA-QG-A5Z2-01 | 952 | 0 | -0.14654 |
| TCGA-AA-3494-01 | 31 | 0 | -0.12757 |
| TCGA-AA-3543-01 | 30 | 0 | 0.20362 |
| TCGA-AA-A01C-01 | 457 | 0 | 0.070303 |
| TCGA-CK-4952-01 | 475 | 0 | 0.891247 |
| TCGA-D5-6531-01 | 540 | 0 | 0.40163 |
| TCGA-AA-A00A-01 | 1157 | 0 | 0.047438 |
| TCGA-D5-5538-01 | 1007 | 1 | 0.123122 |
| TCGA-A6-5660-01 | 888 | 0 | 0.066076 |
| TCGA-AA-3842-01 | 396 | 1 | 0.091755 |
| TCGA-AZ-6605-01 | 159 | 0 | 0.558911 |
| TCGA-CM-5349-01 | 915 | 0 | 0.163496 |
| TCGA-AU-3779-01 | 441 | 0 | 0.454786 |
| TCGA-A6-6137-01 | 824 | 0 | 0.187008 |
| TCGA-F4-6854-01 | 16 | 0 | -0.00385 |
| TCGA-AA-3675-01 | 1431 | 0 | 0.155935 |
| TCGA-NH-A6GA-01 | 6 | 1 | 0.695871 |
| TCGA-AA-A01Z-01 | 1126 | 1 | 0.126086 |
| TCGA-CM-6172-01 | 335 | 0 | -0.00459 |
| TCGA-5M-AATE-01 | 810 | 1 | 0.274889 |
| TCGA-D5-6928-01 | 354 | 0 | 0.228801 |
| TCGA-DM-A1D9-01 | 4270 | 0 | -0.20449 |
| TCGA-AA-A01I-01 | 943 | 0 | 0.193578 |
| TCGA-D5-6930-01 | 406 | 0 | 0.38707 |
| TCGA-AA-3555-01 | 91 | 1 | 0.506487 |
| TCGA-CM-6676-01 | 337 | 0 | 0.732444 |
| TCGA-A6-4107-01 | 987 | 0 | 0.01376 |
| TCGA-AA-A01R-01 | 608 | 1 | 0.358028 |
| TCGA-G4-6294-01 | 517 | 1 | 0.043326 |
| TCGA-AZ-4684-01 | 411 | 1 | 0.122677 |
| TCGA-AA-A01X-01 | 791 | 1 | 0.278704 |
| TCGA-CM-4750-01 | 244 | 0 | 0.360253 |
| TCGA-AA-A01K-01 | 943 | 0 | 0.057999 |
| TCGA-G4-6314-01 | 38 | 1 | 0.879717 |
| TCGA-CA-5796-01 | 377 | 0 | -0.08431 |
| TCGA-AZ-6599-01 | 206 | 0 | 1.380145 |
| TCGA-AD-6901-01 | 468 | 1 | 0.498842 |
| TCGA-G4-6299-01 | 2268 | 0 | 0.464145 |
| TCGA-F4-6808-01 | 1024 | 0 | 0.314327 |
| TCGA-AA-3516-01 | 396 | 0 | 0.147485 |
| TCGA-A6-5666-01 | 509 | 1 | 0.197799 |
| TCGA-AA-3510-01 | 1946 | 0 | 0.059061 |
| TCGA-G4-6293-01 | 4051 | 0 | -0.05469 |
| TCGA-AD-6895-01 | 763 | 0 | 1.056864 |
| TCGA-AA-A02E-01 | 90 | 0 | 0.113259 |
| TCGA-DM-A28A-01 | 805 | 0 | 0.65174 |
| TCGA-A6-6654-01 | 726 | 0 | 0.503058 |
| TCGA-CA-5797-01 | 383 | 0 | 0.421971 |
| TCGA-CK-6747-01 | 820 | 0 | 0.256223 |
| TCGA-AA-A01Q-01 | 31 | 0 | -0.02317 |
| TCGA-AA-3848-01 | 306 | 0 | 0.086447 |
| TCGA-AZ-6606-01 | 357 | 0 | 0.222313 |
| TCGA-AZ-4308-01 | 3324 | 0 | 0.337784 |
| TCGA-AA-A00K-01 | 549 | 0 | 0.200383 |
| TCGA-AA-3534-01 | 882 | 0 | 0.064704 |
| TCGA-AA-A02R-01 | 670 | 0 | 0.199203 |
| TCGA-CM-5861-01 | 335 | 1 | 0.604506 |
| TCGA-AA-3502-01 | 1065 | 0 | 0.024893 |
| TCGA-AA-3697-01 | 2587 | 0 | 0.041077 |
| TCGA-DM-A1HA-01 | 2600 | 0 | 0.504238 |
| TCGA-AA-A017-01 | 457 | 0 | 0.045008 |
| TCGA-CM-6170-01 | 457 | 0 | 0.768208 |
| TCGA-AA-3542-01 | 395 | 0 | 0.114852 |
| TCGA-AA-3812-01 | 762 | 1 | 0.164895 |
| TCGA-AZ-6607-01 | 97 | 0 | 0.679323 |
| TCGA-G4-6323-01 | 419 | 0 | -0.17367 |
| TCGA-AA-3971-01 | 489 | 0 | 0.002205 |
| TCGA-F4-6460-01 | 313 | 1 | 0.988007 |
| TCGA-A6-A56B-01 | 1678 | 1 | 0.16558 |
| TCGA-AA-A00E-01 | 913 | 0 | 0.062147 |

**Supplementary Table 3. Riskscore of 427 enrolled patients’ relapse model.**
