# Supplementary material for: NR4A1 as a potential therapeutic target in colon adenocarcinoma: a computational analysis of immune infiltration and drug response
Source: Front Genet. 2023 Jul 26;14:1181320. doi: 10.3389/fgene.2023.1181320 (PMC10410285; doi:10.3389/fgene.2023.1181320)
Supplement: Supplementary file 2 [file Image1.PDF]

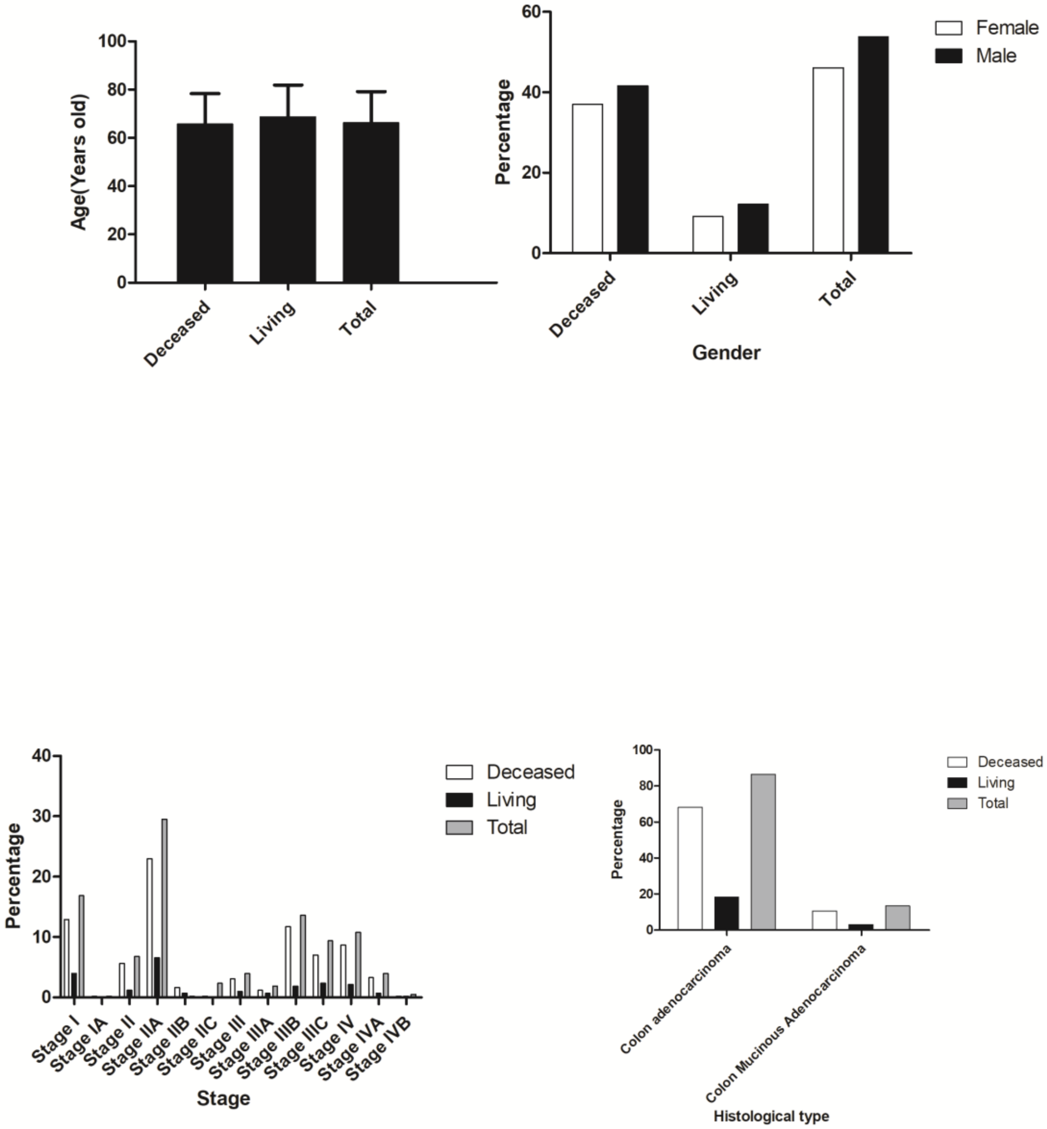

S Fig 1 Bar plot of the clinicopathological characteristics of the 427 patients

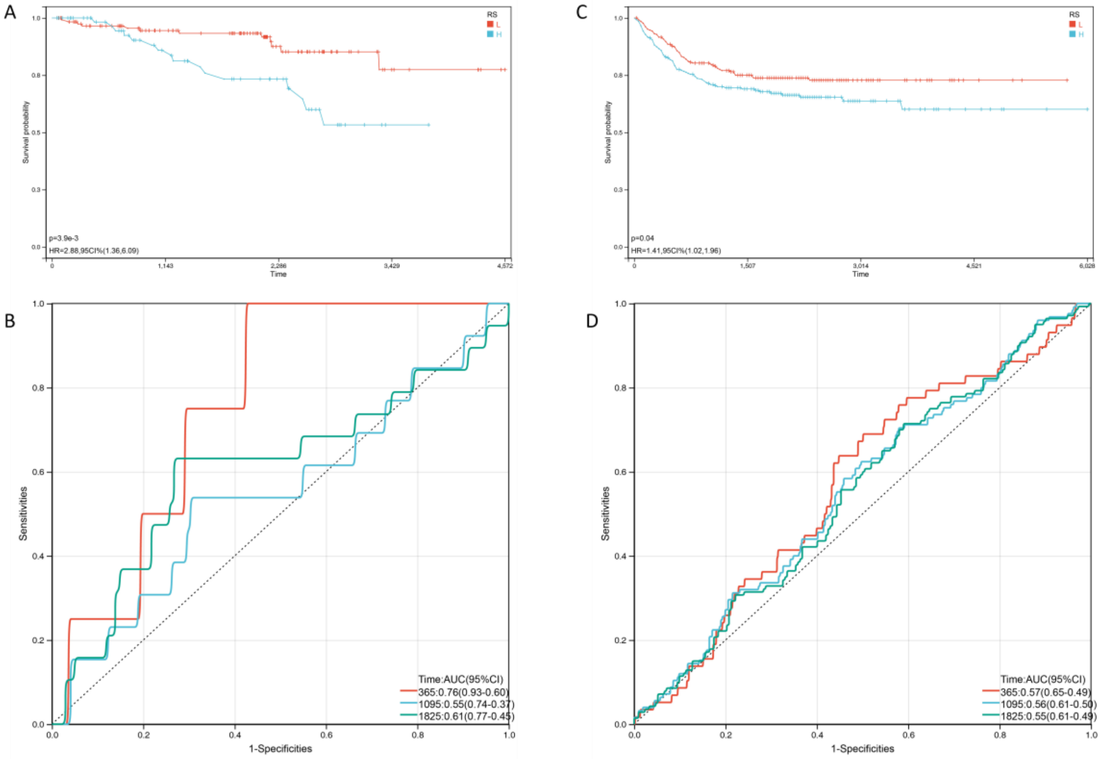

S Fig 2 External validations of overall survival model and relapse model.

A. KM curves of the overall survival riskscore in external dataset.
B. ROC curves of the overall survival in external dataset.
C. KM curves of the relapse riskscore in external dataset.
D. ROC curves of the relapse in external dataset.

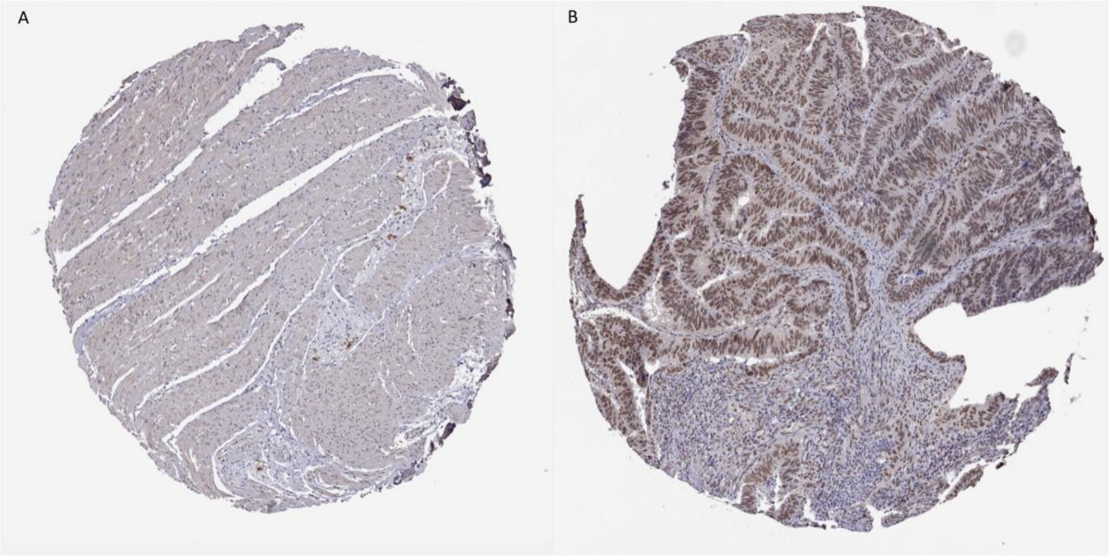

S Fig 3 Protein staining of NR4A1

A. Protein staining of NR4A1 in normal colon samples.
B. Protein staining of NR4A1 in colon adenocarcinoma samples.
